# Major changes of cell function and toxicant sensitivity in cultured cells undergoing mild, quasi-natural genetic drift

**DOI:** 10.1007/s00204-018-2326-5

**Published:** 2018-10-08

**Authors:** Simon Gutbier, Patrick May, Sylvie Berthelot, Abhimanyu Krishna, Timo Trefzer, Mehri Behbehani, Liudmila Efremova, Johannes Delp, Gerhard Gstraunthaler, Tanja Waldmann, Marcel Leist

**Affiliations:** 1 Department for in vitro toxicology and biomedicine (Doerenkamp-Zbinden chair), University of Konstanz, PO Box M657, 78457 Konstanz, Germany; 2 Luxembourg Centre for Systems Biomedicine, University of Luxembourg, Esch-sur-Alzette, Luxembourg; 3 Division of Physiology, Innsbruck Medical University, Schöpfstraße 41/1, 6020 Innsbruck, Austria

**Keywords:** Human genome, Cell stability, Dopamine transporter, genome comparison, genotype-phenotype correlation

## Abstract

Genomic drift affects the functional properties of cell lines, and the reproducibility of data from in vitro studies. While chromosomal aberrations and mutations in single pivotal genes are well explored, little is known about effects of minor, possibly pleiotropic, genome changes. We addressed this question for the human dopaminergic neuronal precursor cell line LUHMES by comparing two subpopulations (SP) maintained either at the American-Type-Culture-Collection (ATCC) or by the original provider (UKN). Drastic differences in susceptibility towards the specific dopaminergic toxicant 1-methyl-4-phenylpyridinium (MPP+) were observed. Whole-genome sequencing was performed to identify underlying genetic differences. While both SP had normal chromosome structures, they displayed about 70 differences on the level of amino acid changing events. Some of these differences were confirmed biochemically, but none offered a direct explanation for the altered toxicant sensitivity pattern. As second approach, markers known to be relevant for the intended use of the cells were specifically tested. The “ATCC” cells rapidly down-regulated the dopamine-transporter and tyrosine-hydroxylase after differentiation, while “UKN” cells maintained functional levels. As the respective genes were not altered themselves, we conclude that polygenic complex upstream changes can have drastic effects on biochemical features and toxicological responses of relatively similar SP of cells.

**Electronic supplementary material:**

The online version of this article (10.1007/s00204-018-2326-5) contains supplementary material, which is available to authorized users.

## Introduction

Cell lines, as biological material essential for many research fields, are difficult to define and standardize (Hartung and Leist 2008; Nims and Reid 2017). Methods like short tandem repeat quantification (STR) can show whether two cells are derived from the same donor (Bian et al. 2017), and classic cytogenetic approaches can provide data on genome integrity at the level of chromosomes or large fragments thereof. Sequencing methods that map individual base pairs (bp) additionally identify small copy number variations (CNVs; 1 kbp–3 Mbp), larger structural variants (SV; > 3 Mbp) and mutations or single nucleotide variants (SNV). Such genome alterations continuously occur in somatic cells at relatively high frequency (Gore et al. 2011; Laurent et al. 2011; Milholland et al. 2017; Youssoufian and Pyeritz 2002), and may often be without functional consequences. This background “genetic noise” makes it difficult to identify changes that affect biological features of interest. Distinguishing relevant changes from background noise of genome alternations is important to the question of whether two subpopulations (SP) of cells are functionally identical. Many fields in fundamental and applied sciences, where cells are the essential material to derive information or to generate bio-products, strongly rely on defining methods for determining identity of the used cells.

Even with apparently clear cell identity established (e.g. by STR profiling), large genetic variations, such as amplification or loss of chromosomal parts, may go undetected, and apparently identical cells may indeed be genetically diverse (Kleensang et al. 2016; Milholland et al. 2017). For instance, some pluripotent stem cell lines tend to develop aneuploidy or translocations during prolonged culture: trisomy 12 or X, and amplification of 17q provide selection advantages by multiplication of stemness or anti-apoptotic genes (Baker et al. 2016; Martins-Taylor and Xu 2012). Also, it is well established that for cell lines like HeLa, many genetically different SP exist (Frattini et al. 2015). On an even more subtle level, cell identity may be affected by small mutations, and SNP profiling is one of the approaches to document such changes (Miller et al. 2014). Next-generation sequencing methods opened the possibility for a closer genetic characterization [by whole-genome sequencing (WGS)] at the bp level (Baker et al. 2016; Krishna et al. 2014; Milholland et al. 2017). By comparing differences in the sequence of a cellular genome to the reference genome, single nucleotide variants (SNV; small deletions, insertions or base exchanges in the range of few nucleotides), CNV (sequence parts larger than 1000 bps that show an average ploidy of > 2 or < 2) and other changes can be detected. Typically, around 3.5–4 million SNVs may be detected for iPSC cell lines compared to the reference genome. After removal of already known SNVs, 1000–2000 SNVs are typically identified between cell lines. These are predominantly heterozygous, and only 5–12 are then expected in coding regions of genes (Cheng et al. 2012; Gore et al. 2011). Notably, also genome changes that do not alter the protein structures may have drastic functional consequences. For instance, CNVs have been associated with neurological and psychiatric disorders (Cai et al. 2015; McConnell et al. 2013; Stefansson et al. 2014).

Studies using WGS point out two factors that favor generation of genetically distinct subpopulations (SP) upon prolonged culture. The first relates to genetic heterogeneity of starting cultures, containing genetically distinct subpopulations (Cai et al. 2014). Second, genetic variants might arise de-novo during sub-culturing and be selected by further propagation of the cell line. In neuronal precursor cells, a potential additional mechanism responsible for the genome dynamics are L1 retrotransposons, which are highly active during neurodevelopment (Coufal et al. 2009), and may cause insertional mutagenesis.

LUHMES cells, examined in this study, are neural precursor cells of the mesencephalon, obtained from an 8-week-old fetus. They were conditionally immortalized with a tetracycline-controlled v-myc transgene (Lotharius et al. 2002, 2005). Its down-regulation triggers the differentiation to electrically active dopaminergic, fully post-mitotic neurons (Scholz et al. 2011). LUHMES have been deposited in 2006 at the American Type Culture Collection (ATCC) (Reid 2017), and a recent report using the banked cells (Zhang et al. 2014) found that they tolerate up to 60 µM of the neurotoxicant 1-methyl-4-phenylpyridinium (MPP^+^), while cells from the original provider laboratory (UKN) died at 3–5 µM (Krug et al. 2014; Poltl et al. 2012; Schildknecht et al. 2009). This large shift in toxicological properties called for a comparative investigation of the cells. As strategies to define functional cell identity are of vast general importance, we explored WGS as a novel approach (Kaas et al. 2015; Seim et al. 2017). Previous studies addressing genetic drift focused mainly on the genome changes arising during prolonged culture. Only little quantitative information is available on how such genetic drift effects cell function. We addressed here the question on whether genome analysis can comprehensively and unambiguously define cell identity relative to some well-defined functions. Our results suggest that a prediction of functionality requires more than the genome data, and that cell identity definition needs to follow a fit-for-purpose definition, which mostly requires specifically adopted analytical endpoints.

## Materials and methods

### Chemicals

Dibutyryl-cAMP (cAMP), fibronectin, Hoechst dye H-33342, resazurin sodium salt, tetracycline, tetramethylrhodamine ethyl ester (TMRE) and MPP^+^ were from Sigma (Steinheim, Germany). Recombinant human FGF-2 and recombinant human glial cell derived neurotrophic factor (GDNF) were from R&D Systems (Minneapolis). Tween-20 and sodium dodecyl sulfate (SDS) were from Roth (Karlsruhe, Germany). All culture reagents were from Gibco/Fisher scientific (Hampton, New Hampshire, USA) unless otherwise specified.

### Cell culture

Handling of LUHMES human neuronal precursor cells was performed exactly as previously described in detail (Schildknecht et al. 2013; Scholz et al. 2011). Briefly, maintained in a 5% CO_2_/95% air atmosphere at 37 °C in proliferation medium (PM), consisting of advanced DMEM/F12 with 2 mM l-glutamine, 1× N2 supplement (Invitrogen), and 40 ng/ml FGF-2, cells were passaged three times a week. For differentiation, 8 million cells were seeded in a Nunclon T175 tissue culture flask in PM. After 24 h, the medium was changed to differentiation medium (DM), consisting of advanced DMEM/F12 supplemented with 2 mM L-glutamine, 1× N2, 2.25 µM tetracycline (to switch off the transgene), 1 mM dibutyryl 3′,5′-cyclic adenosine monophosphate (cAMP) and 2 ng/ml GDNF. 48 h later, cells were trypsinized, and seeded in a density of 1.5 × 10^5^ cells/cm^2^ on dishes precoated with 50 µg/ml poly-l-ornithine (PLO) and 1 µg/ml fibronectin in DM. On day 4 of differentiation (d4), the medium was exchanged for fresh DM.

### Cell viability measurement

*Resazurin* Metabolic activity was detected by a resazurin assay (Schildknecht et al. 2009). Briefly, resazurin solution was added to the cell culture medium to obtain a final concentration of 10 µg/ml. After incubation for 30 min at 37 °C, the fluorescence signal was measured at an excitation wavelength of 530 nm, using a 590 nm long-pass filter to record the emission. Fluorescence values were normalized by setting fluorescence values of untreated wells as 100%.

*LDH release* LDH activity was detected separately in the supernatant and cell homogenate as described earlier (Latta et al. 2000). The ratio of LDH_supernatant_/LDH_supernatant+ cell lysate_ was calculated and expressed in percent (Latta et al. 2000).

### Neurite area detection

Labeling live cells was performed with 1 µM calcein-AM/1 µg/ml H-33342 for 30 min at 37 °C. Images were collected in two different fluorescent channels using an automated microscope (Array-Scan VTI HCS Reader, Thermo Fisher, PA, USA) with high content imaging software (vHCS SCAN, Thermo Fisher, PA, USA). For visualization, an Olympus IX81 inverted epifluorescence microscope with a 20× objective was used. Nuclei were automatically identified in channel 1 (365 ± 50/461 ± 15 nm) as objects according to their size, area, shape, and intensity. The calcein signal was detected in channel 2 (475 ± 40/525 ± 15 nm). An algorithm quantified all calcein positive cells as viable and nuclei stained by H-33342 only as “non viable” cells.

For quantification of the neurite area of d3 cells a well-established algorithm was applied (Stiegler et al. 2011). For d6 LUHMES, cells were fixed and stained for β-III-tubulin and H-33342, and then the same algorithm was applied.

### ATP determination

To determine intracellular ATP, cells grown in 24-well plates were scratched and sonicated in PBS-buffer and boiled at 95 °C for 10 min followed by centrifugation at 10,000*g* for 5 min for the removal of cell debris (Volbracht et al. 1999, 2001). For the detection of ATP levels, a commercially available ATP assay reaction mixture (Sigma, Steinheim, Germany), containing luciferin and luciferase, was used. 50 µl sample and 100 µl of assay-mix were added to a black 96-well plate. Standards were prepared by serial dilutions of ATP disodium salt hydrate (Sigma, Steinheim, Germany) to obtain final concentrations ranging from 1000 nM to 7.8 nM.

### GSH determination

For glutathione determination cells were washed with PBS and lysed in 400 µl of 1% sulfosalicylic acid (w/v). The lysates were collected, sonicated 5 times and centrifuged at 12,000×*g* for 5 min at 4 °C to remove cell debris. Total glutathione content was determined by a DTNB [5,5′-dithiobis(2-nitrobenzoic acid)] reduction assay. 20 µl sample was mixed with 180 µl assay mixture containing 300 µM DTNB, 1 U/ml glutathione-reductase, 400 µM NADPH, 1 mM EDTA in 100 mM sodium phosphate buffer, pH 7.5 (all Sigma, Steinheim, Germany). DTNB reduction was measured photometrically at 405 nm in 5 min intervals over 30 min. GSH standard curves were performed by serial dilutions ranging from 1000 nM to 7.8 nM, respectively.

### Western blot analysis

Cells were lysed in RIPA-buffer (50 mM Tris-base, 150 mM NaCl, 1 mM EDTA, 0.25% sodium deoxycholate, 1% NP40, 1 mM Na3VO4, 50 mM NaF, pH 7.5) containing 1× protease inhibitor (Roche) and 0.5% phosphatase inhibitor cocktail 2 (Sigma, Steinheim, Germany). Determination of protein concentration was performed using a BCA protein assay kit (Pierce/Thermo Fisher Scientific, Rockford, IL, USA). Thirty-five µg of total protein were loaded onto 12% SDS gels. Proteins were transferred onto nitrocellulose membranes (Amersham, Buckinghamshire, UK). Loading and transfer were checked by brief Ponceau staining. Washed membranes were blocked with or 5% BSA in TBS–Tween (0.1%) for 1 h. Primary antibodies were incubated at 4 °C overnight. Following washing steps with TBS–Tween (0.1%), horseradish peroxidase-conjugated secondary antibodies were incubated for 1 h at RT. For visualization, ECL Western blotting substrate (Pierce/Thermo Fisher Scientific, Rockford, IL, USA) was used (Gerhardt et al. 2001).

### Immunocytochemistry

Cells were grown on 13 mm glass coverslips (Menzel, Braunschweig, Germany) in 24-well plastic cell culture plates (NunclonTM) and fixed with 4% paraformaldehyde (Hansson et al. 2000; Hirt et al. 2000). After incubation with the primary antibody overnight (see Table. S1) and with the appropriate secondary antibody for 1 h, Hoechst-33342 (1 µg/ml) was added for 10 min prior to the final washing step. Coverslips were mounted on glass slides with Fluorsave reagent (Calbiochem/Merck/Darmstadt, Germany).

### EdU incorporation

Cells were grown and differentiated on coated glass coverslips and fixed with PBS, 4% paraformaldehyde, 2% sucrose for 15 min. For EdU-staining cells were incubated with 10 µM EdU 30 min previous to fixation. To detect EdU incorporation the Click-iT^®^EdU Alexa Fluor^®^555 Imaging Kit from Life Technologies (Carlsbad, CA, USA) was used as described by the provider. For the quantitative image analysis, we used KNIME Image Processing Extension (Version 1.1.2). KNIME (Konstanz Information Miner) (https://www.knime.com/) is a user-friendly and comprehensive open-source data integration, processing, analysis, and exploration platform designed to handle large amounts of heterogeneous data. The Image Processing Extension (http://tech.knime.org/community/image-processing) of KNIME allows the documentation of the Image Analysis steps in a so-called workflow. Total nuclei and EdU positive nuclei were counted automatically.

### Quantitative real-time PCR (qPCR)

For reverse transcription quantitative PCR analysis, RNA was extracted with the PureLink RNA mini Kit (Invitrogen, Darmstadt, Germany) according to the manufacturer’s instructions. For transcript analyses of LUHMES, primers (Eurofins MWG Operon, Ebersberg, Germany) were designed using AiO (All in One) bioinformatics software (Karreman 2002) and can be found in Table S2. All RT-qPCRs were based on the SsoFast EvaGreen detection system and were run in a CFX96 Cycler (Biorad, München, Germany) and analyzed with Biorad iCycler software (Balmer et al. 2012; Zimmer et al. 2011a). The threshold cycles (*C*_t_) were determined for each gene and gene expression levels were calculated as relative expression compared to GAPDH (2^−(Δ*C*t)^) or as fold change relative to control (2^−(ΔΔ*C*t)^). Δ*C*_t_ and ΔΔ*C*_t_ were calculated using the following formulas:

- $$\Delta {C_{\text{t}}}{\text{ }}={\text{ }}{C_{\text{t}}}\left( {{\text{condition}}\;{\text{X}}/{\text{gene Y}}} \right){\text{ }}-{\text{ }}{C_{\text{t}}}\left( {{\text{condition}}\;{\text{X}}/{\text{GAPDH}}} \right)$$

$$\Delta \Delta {C_{\text{t}}}{\text{ }}={\text{ }}\Delta {C_{\text{t}}}\left( {{\text{condition}}\;{\text{X}}/{\text{gene Y}}} \right){\text{ }}-{\text{ }}\Delta {C_{\text{t}}}\left( {{\text{untreated control}}/{\text{gene Y}}} \right).$$

### Dopamine measurement

Predifferentiated LUHMES cells (d2) were seeded in T75 Nuclon Flask and differentiated according to the protocol. On day 6, cells were lysed in 0.2% Triton X-100, 0.01 M HCl, 1 mM EDTA, 4 mM sodium metabisulfite and 1 mM Ascorbate. Cells were scraped with a cell scraper, sonicated, centrifuged at 3000*g* for 5 min and supernatants were frozen at − 20 °C. The dopamine content in cell lysates was analyzed using Dopamine ELISA Kit (Abnova) according to the manufacturer’s protocol.

### ^3^H-MPP + uptake

Cells were incubated with 5 µM ^1^H-MPP^+^ and 0.125 µCi per 200 µl (15 nM) volume radioactively labeled ^3^H-MPP^+^ at 37 °C for different incubation times. The supernatant and the cells were separated. Cells were washed 3 times in PBS and then mechanically removed from the multiwell plate in an equal volume PBS. 2 ml Ultima Gold AB solution was added to the supernatant and lysed cells. Light emission was measured by Beckman LS 6500 scintillation counter (Schildknecht et al. 2015; Zimmer et al. 2011b).

### Sequencing and genome comparison

DNA was prepared from the “UKN” and “ATCC” LUHMES (ATC CRL-2927) SP using the Gentra Puregene Cell kit (Qiagen; Venlo, Netherlands). After sequencing, the reads (about 30× full genome coverage) were mapped against the NCBI Build 37.2 reference genome (Table S3). The “UKN” SP was paired-end sequenced with Complete Genomics (CG), Inc (Mountain View, CA, USA), using their proprietary sequencing-by-ligation technology (Drmanac et al. 2010). CG performed primary data analysis as part of their Standard Sequencing Service pipeline (v 2.4.0.43), including image analysis, base calling, quality control, mapping and variant calling. The CG pipeline calls different types of variants with independent pipelines including small SNVs and insertions and deletions (indels), copy number variants (CNV), structural variants (SV) and mobile element insertions (MEI). “ATCC” SP was paired-end sequenced (2 × 150 bp) with the Illumina FastTrack Service. Library preparation was performed using the TruSeq Nano DNA kit (Illumina), and processed with the Illumina pipeline version v4.0.2 using the Isaac Aligner (6.15.01) for mapping, Isaac Variant Caller (2.1.4) for calling germline SNVs and indels, the Isaac CNV Caller (1.1.0) for germline CNV calls (Raczy et al. 2013), and Isaac SV caller (0.23.1).

### Definitions of calls

The significant differences from the reference genome (= calls) were categorized as follows: CNV were called using coverage data from both WGS and a human reference genome bin-size of ≈ 1 kbp resolution. They were defined as parts of the genome larger than 1000 bp and with an average ploidy of > 2 or < 2. SV were defined as rearrangements in the genome structure that affect a sequence length ranging from 1 kb to 3 Mb, such as large deletions, duplications and inversions. SNV were defined as single nucleotide exchanges. While small insertions and deletions are subsequently called indels (max size: 50 bp). For CG, SNV and indels were derived from the “var file”. CNV calls were taken from cnvSegmentsNondiploidBeta-* file and high-confidence structural variation, SV events were taken from /highConfidenceSvEventsBeta-* file and MEI from the mobileElementInsertionsBeta-* file. For Illumina, SNV and indels, CNV and SV were extracted from the different VCF files.

SNV from both platforms were combined into CG testvariant format and compared using custom perl/python scripts. For CG data, coverage statistics were derived from coverage and coverageRefScore files for each chromosome. Coverage at every base was assessed directly from these files. For Illumina, coverage information was extracted directly from BAM (Binary Sequence Alignment format) files. ANNOVAR (Wang et al. 2010) (v20150322) was used to annotate the SNV, SV and CNV with RefSeq and Ensembl gene annotations.

### Genetic identity analysis of ATCC and UKN LUHMES genomes

To compare the genetic identity of both LUHMES SP, the PLINK software (Purcell et al. 2007) was used. PLINK is designed to identify kinship levels in genomes based on the comparison of chromosome stretches that show identity-by-descent (IBD). IBD DNA segments share a common ancestor, i.e. they are identical-by-state (IBS) and lack recombination events in the corresponding sequence. Two individuals can share 0, 1 or 2 alleles referring then to IBD = 0, IBD = 1 and IBD = 2, respectively. IBD was determined on the basis of common SNVs and small indels from the HapMap project (downloaded from the Broad resource bundle as part of the GATK toolkit: https://www.broadinstitute.org/gatk/guide/article.php?id=1213). The SNVs and indels called in both genomes were used for IBD analysis. As measure of similarity, we used the so-called PI_HAT score, that ranges from 0 (no relationship) to 1 (identity across the genome), and which is calculated from a weighted sum of probabilities for a large number of genome segments to be IBD [PI_HAT = p(IBD = 2) + 0.5* p(IBD = 1)].

### Comparison of CNV and visualization on chromosomal level

For CNV detection the Illumina BAM files, and the CG REF coverage files were screened by the RCP pipeline (Glusman et al. 2015), which compares a given BAM file or CG coverage profile against Reference Coverage Profiles (RCPs) taken from a large set of whole-genome sequences produced either on the Illumina or CG platforms. RCP performs coverage binning with a bin size of 1000 bp, GC correction, normalization using reference coverage profiles and merging into larger segments. Thereby it detects and reports changes in ploidy of the classified fragments. Resulting CNVs were visualized on the chromosomal level using the open source visualization tool (http://db.systemsbiology.net/cgi-pub/genomeMapBlocks.pl).

To identify candidate genes that differ in copy number in their coding regions between the two SP, DGV (Database of Genomic Variants, MacDonald et al. 2014) was used to filter out CNVs (≥ 50% overlap) that are common in the general population. The CNV not annotated in DGV, but occurring in the ATCC or UKN genome were retrieved here.

### Comparison of SNVs, small indels and block substitutions across genomes

To exclude variants arising from technological differences, the data were re-analyzed using an already published filtering strategy (Reumers et al. 2012) allowing better comparison between the two platforms. This reduced the total number of SNV per genome and increased the overall concordance between the two platforms. Post filtering, we subtracted variants present in either one of the above-mentioned platforms and obtained rare events. We filtered this reduced number of variants detected against variants found in one of the following databases: dbSNP 138, 1000 Genome Project, Exome Sequencing Project 6500 and ExAC (Exome Aggregation Consortium). Variants found in one of those databases were considered common or known variants and only the variants, which were not present in any database (called “somatic”), were considered further. To detect differences of potential phenotypic impact, the somatic events were screened for variants potentially affecting splicing and amino acid sequence using the ANNOVAR annotations from RefSeq and Ensembl. The genes annotated to this amino acid changing mutations were sorted for specific occurrence in the “ATCC” or “UKN” genome.

### Pathway over-representation analysis

For overrepresentation analysis, a list of all genes with somatic variants (CNV, SV and SNV) specific for any of the two LUHMES SP was assembled. Using the Ingenuity Pathway Analysis (IPA^®^) software (Kramer et al. 2014), it was examined whether there were pathways in which these genes were overrepresented. The output was sorted according to the *p* value of the over-representation test.

### STR phenotyping

Identity of the used SP was confirmed by STR (Short Tandem Repeat) analysis as described previously (Pallocca et al. 2017). Briefly, DNA samples from the SPs were prepared using a commercial kit (Puregene Cell Kit, Qiagen). The kit GlobalFiler® PCR Amplification Kit (Thermofisher) was then used to determine the cell-specific profile for 16 different genomic loci using an Applied Biosystems GeneMapper Device. STR results showed 100% identity of the profiles of both SP.

### Statistics, data mining and data availability

Cytotoxicity data (ATP, GSH, LDH, resazurin) and qPCR are presented as means of at least three independent experiments, and statistical differences were tested by Student’s t-test or ANOVA with post-hoc tests as appropriate, using GraphPad Prism 5.0 (Graphpad Software, La Jolla, USA). The genomic data of the ATCC CRL-2927 cell line have been deposited in the BioProject database as PRJNA38682. Annotated variant files for CNV, SV and SNV (VCF format) for both SP have been deposited for public access at ELIXIR Luxembourg under (10.17881/LCSB.20180321.01).

## Results

### Similarities and differences in the response to toxicants

**Fig. 1:**
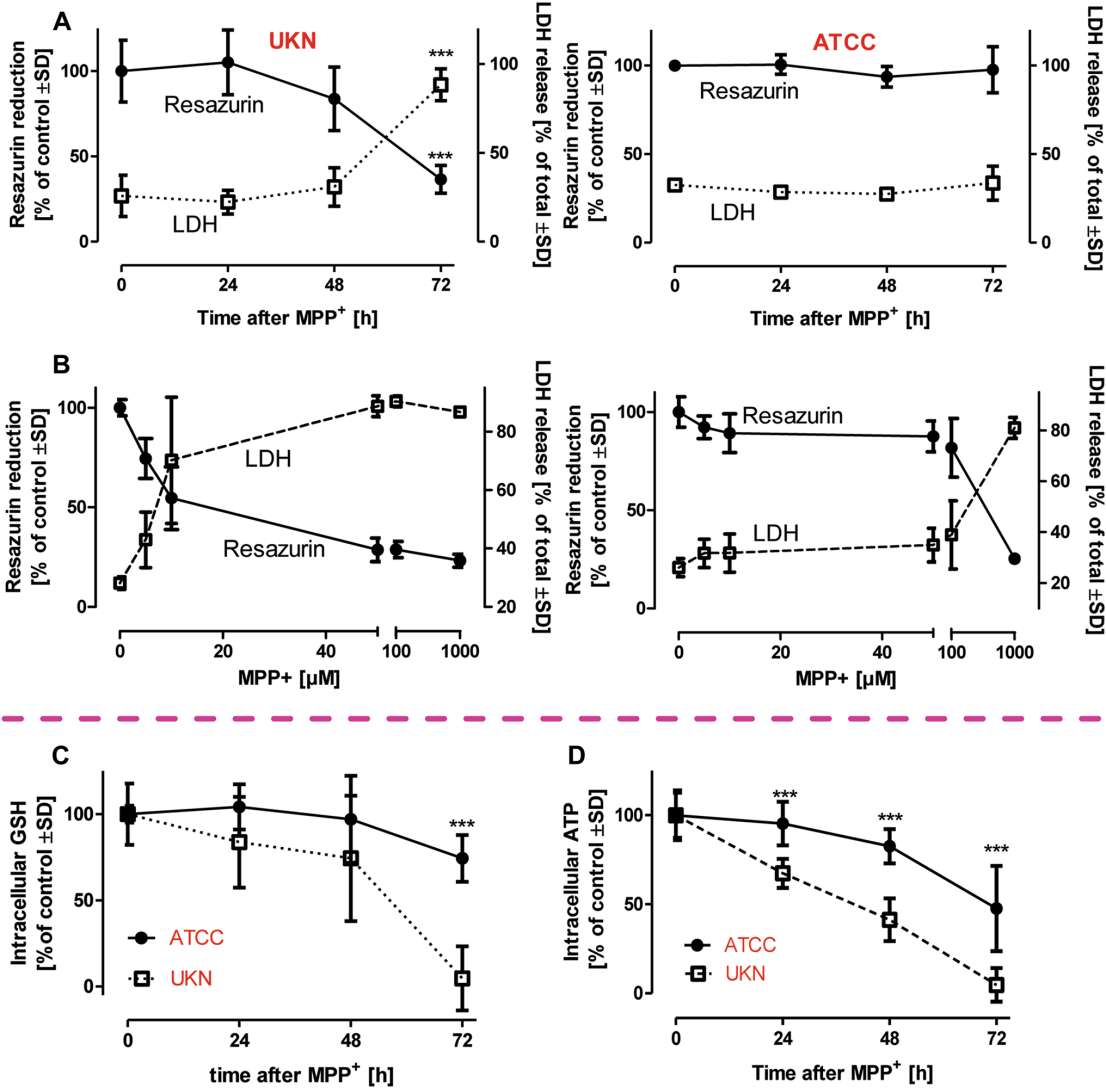
Similarities and differences in the reaction to different toxicants. LUHMES cells from two different sources [University of Konstanz (UKN); American Type Culture Collection (ATCC)] were cultured according to a standard protocol. LUHMES cells were seeded at a density of 1.5 × 10^5^ cells/cm^2^ at d2. Medium was exchanged at d4 and exposed to MPP^+^ starting at d6. **a** Viability was assessed at d9 by measuring resazurin reduction and LDH release after cells have been exposed to MPP^+^ [5 µM] for times as indicated. Data are means ± SD of three independent experiments.***p ≤ 0.001 **b** UKN and ATCC LUHMES were incubated with indicated concentrations of MPP^+^. After 48 h viability was assessed measuring resazurin reduction and LDH release, Data are means ± SD of three independent experiments. **c** Intracellular levels of total glutathione (GSH + GSSG) were measured in cells treated with MPP^+^ [5 µM] for indicated time periods and represent means ± SD of three independent experiments. **d** Intracellular levels of total ATP were measured in cells treated with MPP^+^ [5 µM] for indicated time periods. Data are means ± SD of three independent experiments.***p ≤ 0.001

The two subpopulations (SP) of LUHMES cells, “UKN” and “ATCC” were used for comparative neurotoxicity experiments. As reported earlier, the established Parkinsonian model toxicant MPP^+^ (Schildknecht et al. 2017), at a concentration of 5 µM, triggered cell death in “UKN” but no sign of degeneration in “ATCC” for up to 72 h (Fig. 1a). This was confirmed in an alternative setup, in which “UKN” LUHMES died after 48 h, when exposed to ≥ 5 µM MPP^+^ while the “ATCC” SP tolerated up to 100 µM (Fig. 1b). In line with the observed difference in cell death, “ATCC” LUHMES maintained their pool of the intracellular redox-buffer glutathione for 72 h, when exposed to MPP^+^, while this was severely depleted in “UKN” (Fig. 1c). Essentially similar observations were made for ATP (Fig. 1d), an energy metabolite particularly sensitive to MPP^+^ toxicity.

### Comparison of the genome structures of the two LUHMES SP

Having observed these differences, we tested whether the “UKN” and “ATCC” SP were indeed based on the same cell line. Their STR-profiles were compared, and we found 100% conformity of “UKN” and “ATCC”(Fig. S1). Moreover, this profile agreed with the one provided by ATCC (Fig. S1).

To obtain broader information on the genome status of the cells, whole-genome sequencing (WGS) was performed. Reads were aligned against the human reference genome at a mean coverage of 33.3 × (98.7% of the genome was covered with more than 20× reads) for “UKN”, and of 38.9 × (93.1% of the genome was covered with > 20×) for “ATCC” (Tab. S3).

The first use we made of the WGS data was to establish the kinship-level of the two SP. Using software originally developed for paternity testing [PLINK, (Purcell et al. 2007)] or profiling of tumor heterogeneity, the two genomes were mapped for their “identity by descent” (IBD) and the corresponding PI_HAT score was calculated. The result of 0.9959 is in the range usually found for monozygotic twins or for duplicate tissue samples (Yang et al. 2011). This solidly confirmed the common ancestry of the two SP, and indicated that there was no contamination.

**Fig. 2:**
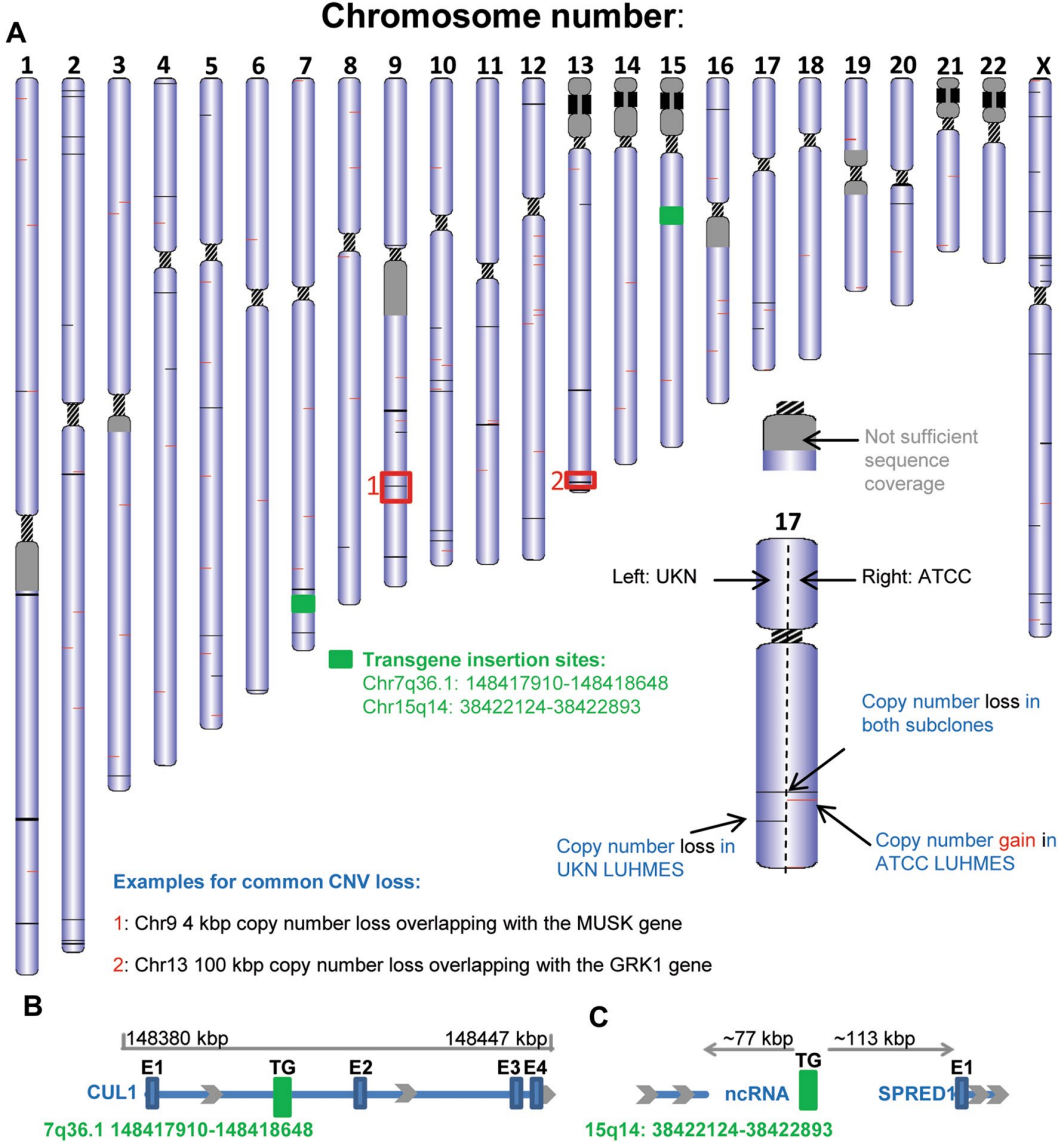
Genome-wide copy number status. LUHMES cells from two different sources [University of Konstanz (UKN); American Type Culture Collection (ATCC)] were sequenced by either complete genomics (UKN) or Illumina (ATCC) sequencing platforms. **a** Copy number variations and transgene insertion sites were depicted on chromosome level. CNVs were visualized on the chromosomal level using the open source visualization tool (http://db.systemsbiology.net/cgi-pub/genomeMapBlocks.pl). Black lines indicate losses and red lines gain in CNV. Transgene insertion sites are marked in green. Examples chosen for detailed resolution are indicated with red boxes and labeled 1 and 2. Detailed resolutions are depicted in Fig. S4. **b** Graphical scheme of transgene insertion site on chromosome 7, which is located within an intron of the Cullin 1 gene. Coordinates are based on the GRCh27 reference genome. **c** Graphical scheme of transgene insertion site on chromosome 15, which is located in the intergenic region

In a next step, the genome data for both cell lines was searched for larger structural rearrangements and copy number changes. Overall chromosomal structure and ploidy were normal for both SP (Fig. 2a). In total, 221 structural variants (SV; typically between 1 kbp and 3 Mbp) not reported in the common genome databases were identified (Fig S2A). Out of these, 15 SV were present in both SP, three were identified only for the “UKN” SP, and the others (containing 179 inversions) were detected only in the “ATCC” LUHMES (Fig. S2A).

We then identified all genes in the proximity of SV (Fig. S2B). This list of candidate factors for altered toxicant sensitivity were examined for reported functions, and none of them offered a simple explanation for the difference of the two LUHMES SP. We, therefore, analyzed in a next step the copy number variations (CNV) as potential candidates. A sizeable number (*n* = 297) of non-common CNV was identified, some of them SP specific (Fig. 2a). After filtering for overlap with protein-coding regions, 16 CNV were found only in “UKN” cells and 35 only in “ATCC” cells (Fig. S3). As expected, both SP shared some (*n* = 19) CNV, as they originate from the same ancestor. Illustrative examples for the latter group are a 4 kbp deletion on chromosome 9, located within the Muscle Associated Receptor Tyrosine Kinase (MUSK) gene, or a 100 kbp deletion on chromosome 13 located within the G Protein-Coupled Receptor Kinase 1 (GRK1) gene (Fig. 2a; Fig. S 4A + B).

Moreover, both cell lines had the same two transgene insertion sites (Fig. 2a), which were mapped to the intronic regions of the cullin 1 (Cul1) gene on chromosome 7 (Fig. 2b) and an intergenic region of chromosome 15 (Fig. 2c).

### Single nucleotide variants (SNV) affecting protein amino acid sequence

**Fig. 3:**
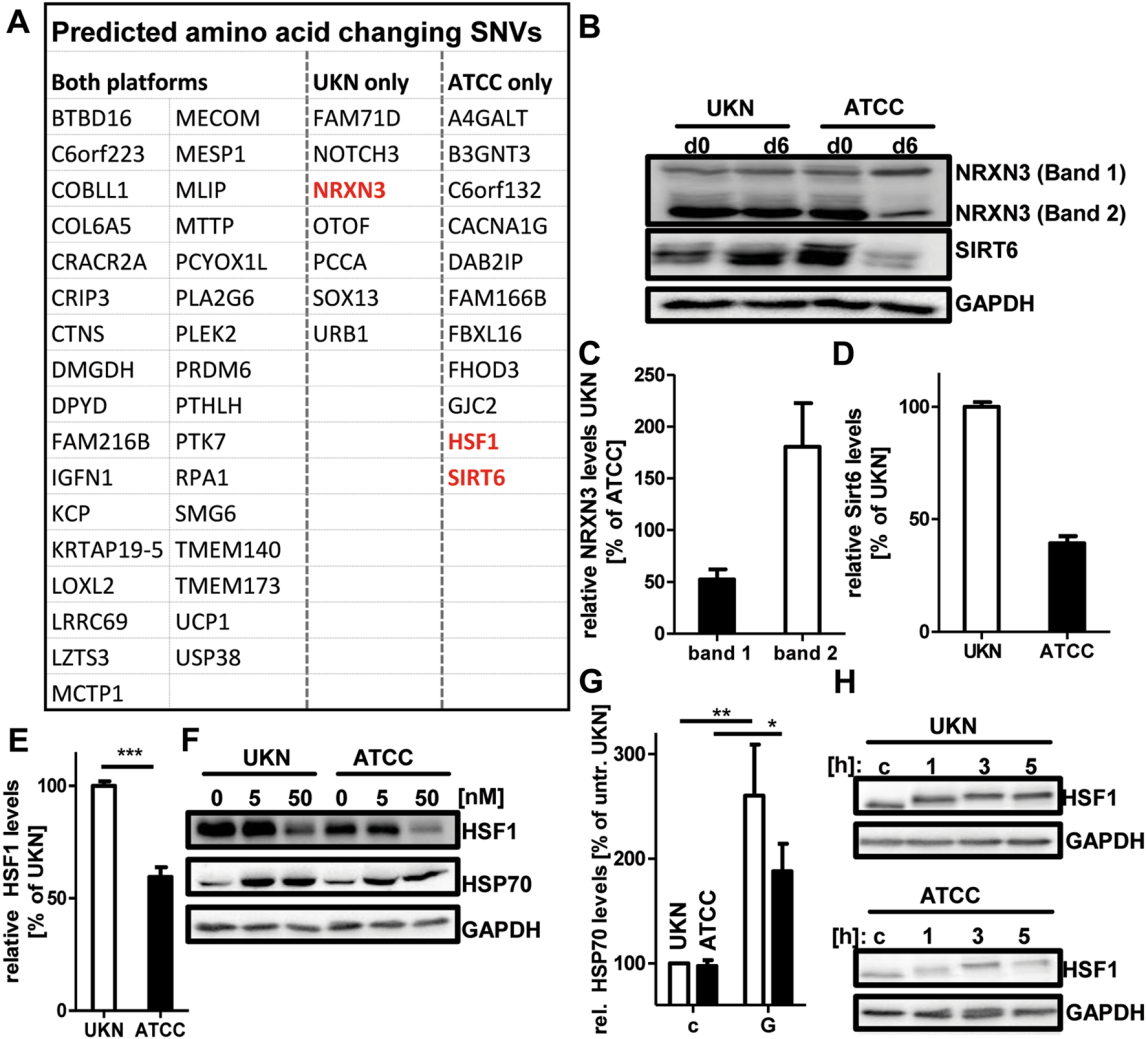
Amino acid changing SNVs. SNVs affecting proteins in LUHMES from two different sources [University of Konstanz (UKN); American Type Culture Collection (ATCC)] were filtered from the total SNVs called as described in the material and methods section in detail. **a** Genes affected by common LUHMES SP and “UKN” or “ATCC” specific amino acid changing SNV. The genes marked in red were further assessed on protein level for differences between the two SP. **b** Undifferentiated (d0) and mature (d6) LUHMES cells were lysed and analysed by Western blot analysis using anti- NRXN3, anti-SIRT6 and anti-GAPDH antibodies. **c, d** Densitometric quantification of band intensities of NRXN3 in d6 cells (**c**) and SIRT6 in d6 cells (**d**). **e** Densitometric quantification of band intensities of HSF1 in d6 cells. Intensities are depicted relative to the intensity in the “UKN” SP. **f** LUHMES (d6) cells of both SP were treated with indicated concentrations of Geldanamycin for 24 h. After incubation cells were lysed and analysed by Western blot using anti-HSF1, anti-HSP70 and anti-GAPDH antibodies. **g** Densitometric quantification of band intensities of HSP70 induction by Geldanamycin. Intensities are depicted relative to the intensity in the untreated “UKN” SP. Band intensities of 5 and 50 nM Geldanamycin were combined. **h** LUHMES (d6) cells of both SP untreated (**c**) and with indicated time after start of heat shock (43° C/1 h). After incubation, cells were lysed and analysed by Western blot using anti-HSF1 and anti-GAPDH antibodies

Altogether about 4.5 million SNV (= small mutations) were called, when the LUHMES genomes were compared to the reference genome (Fig. S5A). These were filtered for known and common variants (Fig. S5 A–C), and quality corrected and filtered for technology biases (Reumers et al. 2012). This resulted in 23,400 LUHMES specific SNV (not occurring in the general population) in LUHMES genomes (Fig. S5B). Further selection for changes predicted to change an amino acid in proteins (Fig. S5C) resulted in a final set of 29 protein changing variants (Fig. 3a) that differed between “UKN” and “ATCC” LUHMES, and 33 rare variants found in both LUHMES SP (Fig. S5C).

From the group of differential mutations, we selected candidates (Fig. 3a, marked in red) that might be responsible for toxicological differences observed between the SP (based on their known biological role). Western blotting was used to investigate altered expression levels of NRXN3 (neural cell adhesion molecule), HSF1 (master controller of the heat shock response), and SIRT6 (cell stress and progeria-associated protein deacetylase). For these three candidate genes, we observed indeed different protein levels in neuronally differentiated LUHMES (Fig. 3b–f). NRXN3 is known for extensive alternative splicing (Rowen et al. 2002), and the ratio of its major bands differed strongly in “ATCC” vs. “UKN” neurons (Fig. 3b, c). SIRT6, although expressed normally in proliferating LUHMES (d0), was strongly down-regulated in d6 “ATCC”, compared to d6 “UKN” (Fig. 3b, d). Moreover, HSF1 was found at lower levels in “ATCC” compared to “UKN” (Fig. 3e, f). To test for functional implications of this finding, cells were exposed to the HSP90 inhibitor geldanamycin. As expected, HSF-1 was down-regulated in both SP. At the same time, a counter-regulation of HSP70 was observed in both SP (Fig. 3f, g). This suggests that the mutation in HSF1 (“ATCC”) did not abolish the overall function of this transcription factor, but it affected its overall cellular expression levels (Fig. 3e, f). This was also confirmed when cells were exposed to heat stress. As described earlier (Budzynski et al. 2015) this leads to a rapid post-translational modification (higher apparent MW) of HSF-1, and this biochemical feature was observed in both SP (Fig. 3h). These findings provide proof of principle that genetic differences between the LUHMES SP, identified by WGS, lead to biochemically measurable alterations. Some of the identified genes are involved in controlling cellular resilience (e.g. HSF1) or aging/degeneration (SIRT6). To explore the functional implication of many potential candidates with respect to MPP^+^ toxicity in LUHMES appeared to be impossible within a reasonable time span. We performed, therefore, additional toxicity experiments to narrow down the list of genes with matching biological properties. Rotenone has the same cellular target as MPP^+^ (Terron et al. 2018), colchicine is general inducer of neuronal apoptosis (Volbracht et al. 1999), and antimycin A /DETA-NONOate trigger different types of oxidative stress and mitochondrial inhibition. They all showed the same toxicity to “ATCC” and “UKN” LUHMES (Fig. S6). These findings suggest that genes involved in general cell death regulation do not account for the differences observed for MPP^+^ (Fig. 1).

### Detailed phenotypic characterization

**Fig. 4:**
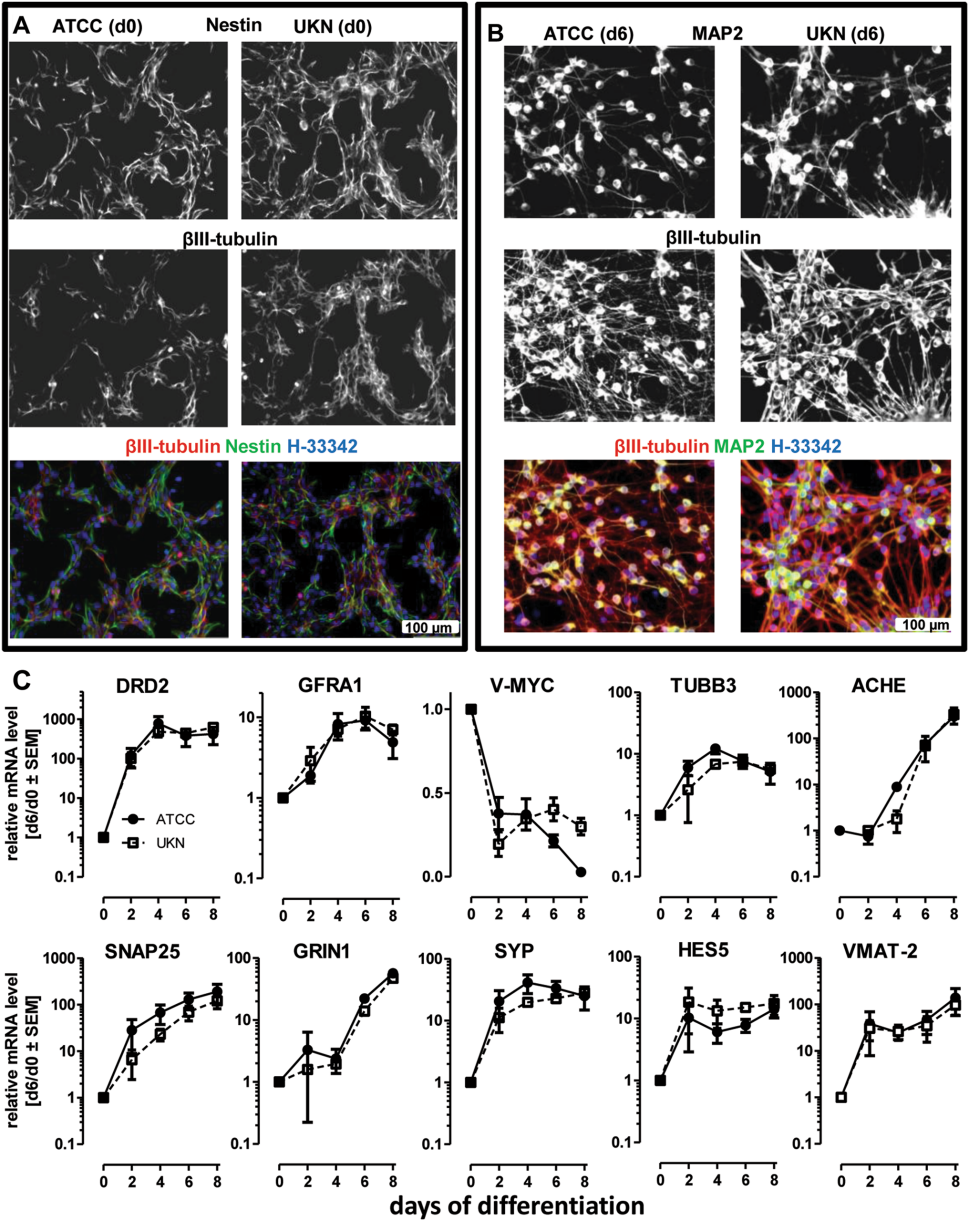
Similarities in neuronal differentiation. LUHMES cells from two different sources [University of Konstanz (UKN); American Type Culture Collection (ATCC)] were cultured and differentiated according to the published standard protocol. **a** Morphology and structural properties of undifferentiated LUHMES (d0) cells (of both SP) were assessed by immunocytochemistry staining for β-III tubulin, Nestin and H-33342. **b** Morphology and structural properties of differentiated LUHMES (d6) cells (of both SP) seeded at a density of 0.5 × 10^5^ cells/cm^2^ were assessed by immunostaining for β-III tubulin (red), MAP2 (green) and H-33342 (blue). **c** LUHMES cells were differentiated for the indicated time points (0; 2; 4; 6; 8 days) and mRNA samples were prepared Changes in gene expression of the neurodevelopmental marker genes DRD2, GFRA1, v-MYC, TUBB3; ACHE, SNAP25, GRIN1, SYP, HES5 and VMAT-2 of LUHMES cells from both SP were monitored by qPCR. Values are expressed relative to expression levels in undifferentiated LUHMES cells. Data are means ± SEM for three different experiments. (Color figure online)

The small genetic changes and the resultant biochemical modifications may subtly affect the neuronal differentiation of cells, and therewith their toxicological response to a specific neurotoxicant. Therefore, we undertook a detailed phenotypic characterization. The overall morphology of the two SP was assessed by immunocytochemistry (ICC), and expression of marker genes [evaluated for the initial characterization of “UKN” LUHMES (Scholz et al. 2011)] was quantified by qPCR. Undifferentiated (d0) SP were similar with respect to the patterns of nestin and β-III-tubulin staining (Fig. 4a), and also differentiated cells (d6) appeared to be morphologically similar, as indicated by the distribution of microtubule-associated protein-2 (MAP2), β-III-tubulin and post-synaptic density protein 95 (PSD95) (Fig. 4b; Fig. S7). We compared the changes in gene expression for marker genes (Fig. 4c). No differences between the SP were observed for DRD2, GFRA1, TUBB3, SYP, SNAP25, GRIN1, HES5, VMAT-2 and ACHE, and the down-regulation of the v-myc transgene, upon tetracycline addition, was equally effective in both SP (Fig. 4c). Based on these data, the two SP would be rated as being similar.

**Fig. 5:**
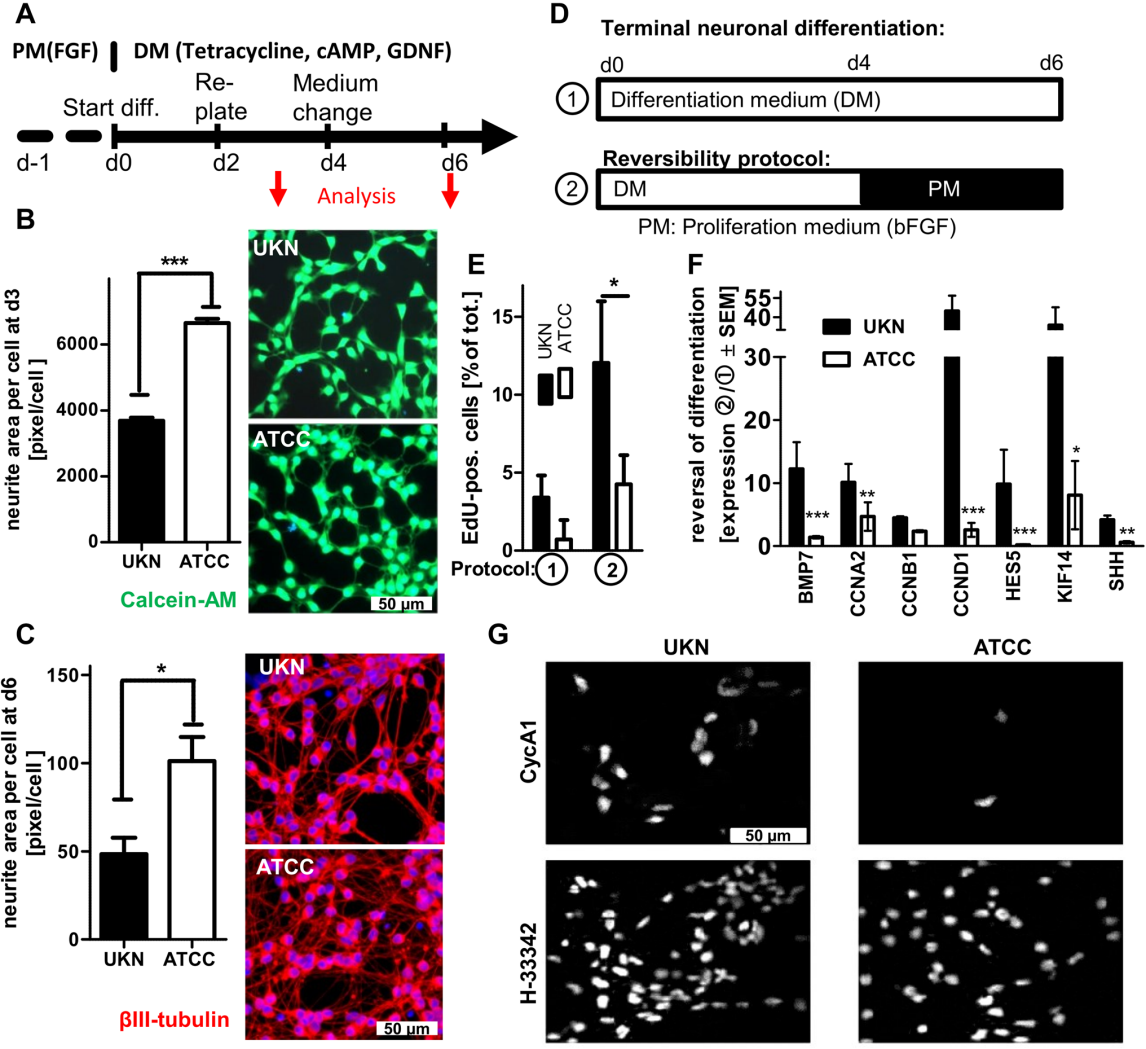
Comparison of neurite outgrowth and cell cycle exit. LUHMES cells from two different sources [University of Konstanz (UKN); American Type Culture Collection (ATCC)] were differentiated according to a published standard protocol. **a** Cells were replated at d-1 and differentiation was started by removal of proliferation medium (PM) and the addition of differentiation medium (DM) containing tetracycline, cAMP and GDNF at d0. Cells were re-plated at a density of 1.5 × 10^5^ cells/cm^2^ at d2. Medium was exchanged at d4 and “mature cells” were ready at d6, e.g. for measurement of the neurite network or toxicant exposure. **b** Cells of both SP were stained on d3 with calcein-AM and H-33342. The neurite area per field was assessed in live cultures by automated microscopy in combination with an algorithm adapted for neurite area quantification (cellomics). In parallel, the number of cells per field was quantified (H-33342 positive nuclei), so that the average area/cell could be calculated. **c** “UKN” and “ATCC” SP were differentiated until d6. These cells were fixed and neurites were assessed by immunocytochemistry staining for β-III tubulin and H-33342. The total neurite area per field (based on β-III-tubulin positive pixels) was measured by automated microscopy in combination with an algorithm adapted for neurite area quantification (cellomics). In parallel, the number of cells per field was quantified, so that the average area/cell could be calculated. **d** LUHMES cells of both SP were cultured in two different cultivation procedures. Therefore medium was exchanged at d4 to either (1) differentiation medium (DM) or (2) proliferation medium (PM). **e** Experiment was stopped at d6 and the EdU positive cells for both culture conditions were quantified using fluorescence microscopy and automated counting. **f** Changes in gene expression in cell cycle-related genes of cells treated with proliferation medium (2) relative to cells differentiated according to the standard protocol (1) were measured at d6 and expressed as the ratio of condition (2)/condition (1). **g** Cell cycle activity of LUHMES cells of both SP cultured according to 2 was assessed by immunocytochemistry staining for cyclin A1 and H-33342. Data are means ± SD for three independent cell differentiations of different passages. ***p ≤ 0.001 **p ≤ 0.01 *p ≤ 0.5

To unravel more subtle differences, we quantified neurite growth (Fig. 5a) as functional endpoint (Krug et al. 2014; Stiegler et al. 2011). Both SP formed an intricate neurite network, but “ATCC” LUHMES displayed a significantly higher neurite area per cell compared to “UKN” LUHMES at day 3 and 6 of differentiation (Fig. 5b, c). This hints to subtle differences in differentiation control between the two SP, possibly linked to signaling factors or transgene activity.

### Differences in the reversal of differentiation

To scrutinize the efficiency of the tet-off system more thoroughly, an experimental setup was developed that triggered neuronal differentiation for 4 days, and then probed, to which extent it could be reversed by re-addition of FGF and withdrawal of GDNF/tetracycline (Fig. 5d). “Reversal” was measured as a number of cells re-entering the cell cycle (EdU incorporation). A higher percentage of EdU positive (reversal) cells was observed in “UKN” compared to “ATCC” (Fig. 5e). This was confirmed by other cell cycle markers, such as Ki67 and CycA1, and it fully agreed with a stronger re-induction of cell cycle genes in “UKN” compared to “ATCC” LUHMES (Fig. 5e, f; Fig. S8A + B). These experiments hinted to a subtle difference in transgene shutoff and differentiation speed between the two SP. As “ATCC” attained the post-mitotic neuronal phenotype faster than “UKN”, the differentiation efficiency does not explain the reduced sensitivity to the neurotoxicant MPP^+^. However, it may have an impact on other types of experiments LUHMES may be used for.

### Differences in the dopaminergic phenotype

Another explanation of the sensitivity differences may be an effect on an upstream regulatory circuit related to the dopaminergic properties of LUHMES. To probe this hypothesis, we performed over-representation analysis of the identified mutations with respect to known signaling and regulation pathways. Amongst the top five most over-represented pathways, we identified “cAMP signaling”, “dopamine receptor signaling” and “tetrahydrobiopterin biosynthesis” in “UKN” LUHMES and “protein kinase A signaling” in “ATCC” LUHMES as potential explanations. These pathways affect the dopaminergic differentiation and the key enzyme TH, and polygenic effects on these regulatory circuits could result in differences in the dopaminergic phenotype (Fig. S9–11).

**Fig. 6:**
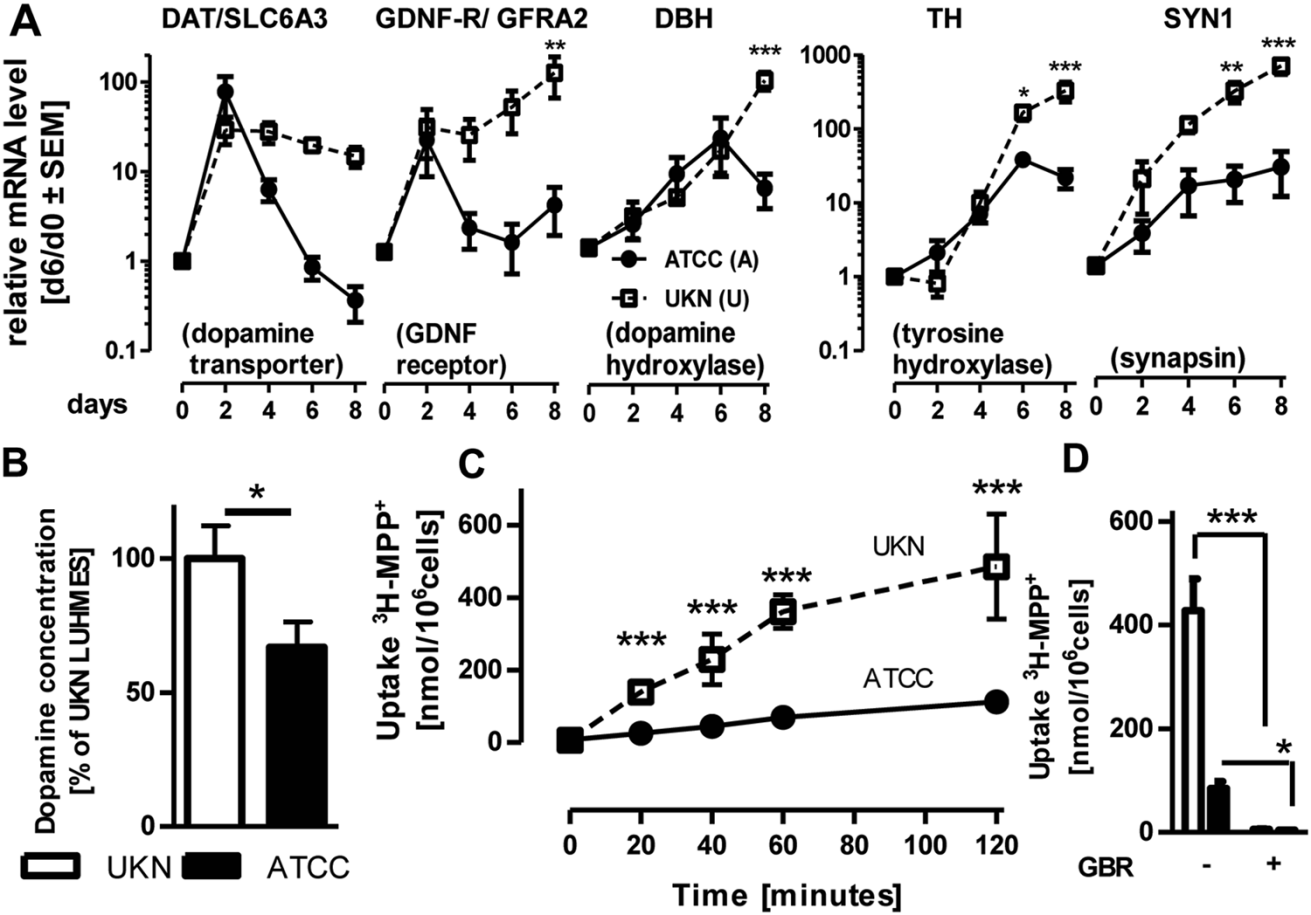
Significantly changed differentiation markers. LUHMES cells from two different sources [University of Konstanz (UKN); American Type Culture Collection (ATCC)] were cultured according to a standard protocol. **a** LUHMES cells were differentiated for the indicated time points (0; 2; 4; 6; 8 days) and mRNA samples were prepared. Changes in gene expression of the neurodevelopmental marker genes DAT, GFRA2, DBH, TH and SYN1 were monitored for both SP. Values are expressed relative to expression levels in undifferentiated LUHMES cells and represent means ± SEM from three independent experiments. ***p < 0.001 **p < 0.01 *p < 0.5. **b** Dopamine content of LUHMES (d6) cells of both SP was analyzed using a Dopamine ELISA Kit (Abnova). **c** Uptake of ^3^H-MPP^+^ in “UKN” and “ATCC” SP was assessed for incubation times as indicated. Therefore, 5 µM MPP^+^ containing 0.3125 µCi ^3^H-MPP^+^ were applied to the cultures and radioactivity in supernatant and cell lysates was measured using a scintillation counter. Data are means ± SD from three independent experiments ***p < 0.001. **d** Uptake of ^3^H-MPP^+^ in “UKN” and “ATCC” LUHMES was measured after 2 h incubation. Therefore, 5 µM MPP^+^ containing 0.3125 µCi ^3^H-MPP^+^ in presence or absence of GBR-12909 [1 µM] were applied to the cultures. Radioactivity in supernatant and cell lysates was measured using a scintillation counter. ***p < 0.001

Considering the results of the pathway enrichment analysis, we wondered whether differences in the dopaminergic phenotype might account for the observed differential toxicity, even though none of the central genes (DAT, TH, VDAC2, AADC, GFRA2, NURR1, LMX1, etc.) appeared to be directly mutated. LUHMES cells have been used as a model for dopaminergic neurons in several studies, and MPP+-toxicity depends on such properties (Delp et al. 2017; Efremova et al. 2015; Lotharius et al. 2005; Zhang et al. 2014). We, therefore, compared the mRNA expression levels of dopaminergic markers during the differentiation. Interestingly, both SP strongly up-regulated the dopamine transporter (DAT) and GDNF receptor (GFRA2) at day 2 of differentiation, but the “ATCC” SP down-regulated these two genes at day 4–8 of differentiation, while they stayed up-regulated in the “UKN” LUHMES (Fig. 6a). Furthermore, there were also significant differences in the regulation of tyrosine hydroxylase (TH), the rate determining enzyme of dopamine synthesis, and few other neuro-specific genes (DBH and SYN1) (Fig. 6a). In line with the lower TH levels, there was a difference in the intracellular dopamine levels, which further confirmed a weaker dopaminergic phenotype of the “ATCC” subclone compared to “UKN” (Fig. 6b). Relative expressions at day 8 differed between the two SP for DAT, GFRA2, DBH and TH (Fig.S12A). Accordingly, TH and DAT protein was abundant in “UKN” LUHMES on d8 but not in the “ATCC” SP (Fig. S12B + C). Moreover, the uptake of MPP^+^ a substrate of the dopamine transporter was significantly higher in “UKN” compared to “ATCC” LUHMES (Fig. 6c). Use of the specific DAT-blocker GBR-129009 confirmed that all MPP^+^ uptake was mediated by the DAT and that this functional difference would impact MPP^+^ toxicity (Fig. 6d).

## Discussion

LUHMES cells are used as highly reproducible test system for neurotoxicity investigations or to model aspects of neurodegenerative disease (Delp et al. 2017, 2018; Harris et al. 2017; Krug et al. 2014; Poltl et al. 2012; Sarkar et al. 2017; Schildknecht et al. 2013; Scholz et al. 2011; Stiegler et al. 2011; Szego et al. 2017; Xiang et al. 2013; Zhang et al. 2014). Since the MPP^+^ model for Parkinson’s disease is frequently used by us and others (Schildknecht et al. 2017; Terron et al. 2018), the finding that the induction of cell death in LUHMES purchased from ATCC required 16 times higher concentration of the neurotoxicant MPP^+^ (Zhang et al. 2014), than found for the original cells (“UKN”) (Krug et al. 2014; Poltl et al. 2012; Schildknecht et al. 2009), raised concerns about the definitions of cell line identity. Even though we found here SP of LUHMES from different sources to be highly similar on the genomic level (same STR-profile; PI_HAT score ≈ 1), we identified clear phenotypic and toxicological differences using a targeted identity profiling. The attenuated dopaminergic phenotype identified in the “ATCC” SP, explained the selectively increased resistance to the Parkinsonian toxicant MPP^+^. The results of this study thus provide compelling evidence that standard measures of cell definition, ranging from phenotypic markers, over STR profiling and even WGS data are not sufficient to define cell identity. Even highly related cell populations have too many differences to be followed up, especially if it is considered that also non-coding variants may play important roles. From disease biology it is well-known that mild (no full loss of any gene) copy number variation can have pronounced effects, and the gene combinations responsible for such effects have been hard to define (Nielsen et al. 2017). Our findings, together with these background considerations, suggest that targeted testing, related to the specific functional demands of cells in a given study, is required.

Here, we successfully showed that the two LUHMES SP are indeed derived from the same cells. Although, this may appear trivial, it is an important confirmation, as a lot of commonly used cell lines are misidentified (e.g. wrong tissue origin or wrong species) or contaminated by other cells (e.g. HeLa)(Dirks and Drexler 2013; Dolgin 2016; Gartler 1968; Hughes et al. 2007; Landry et al. 2013; Pamies et al. 2017; Reid 2017). During the last decades, as cross-contaminations and cellular misidentifications became apparent, the importance of cell line identity gained increasing attention (Dirks and Drexler 2013; Freedman 2015; Freedman et al. 2015b). The rapid development in the field of genome sequencing allowed us now to address questions on genetic drift and stability at higher levels of resolution.

Since we observed very high genetic concordance and two identical transgene insertion sites in each SP, our WGS data confirmed the common clonal origin of the two SP. Nevertheless, we also detected differences on the genome level, which initially appeared to be relatively high in number. This may be in part be due to two different sequencing platforms used here to derive WGS data and the resulting slight differences in genome coverage and calling algorithms. However, after application of established software tools and quality assurance, to correct for the noise generated by the different sequencing platforms (Reumers et al. 2012), the number reduced drastically and was in the range of other reports (Cheng et al. 2012; Gore et al. 2011).

One remaining non-corrected difference is the overrepresentation of inversions in “ATCC”. This comes mainly from the different SV calling method used for the Illumina sequencing data. SVs were here called with a newly developed method called Manta (Chen et al. 2016) that is not available for Complete Genomics data. Therefore, it is not clear, if those inversions are SP specific or even false positive calls. The difference had, however, no influence on eh outcome of this study and on the conclusions drawn.

In other studies, genome profiling has been used mainly to describe cellular differences, but even the most recent large-scale approaches (Grobner et al. 2018; Ma et al. 2018) fail to make predictions from genome changes to cell function (e.g. altered function or subtype of neoplastic disease). This is, however, circumstantial evidence, that long-term culturing, genetic drift and altered functional properties of cells are causally related. It has been shown that sub-culturing with too many passages may affect cell line characteristics and thereby reproducibility. For example, Caco2 cells display different transport and toxicological properties with increasing number of passages (Hughes et al. 2007). Genetic heterogeneity of the parental line and sub-selection during culturing is suspected to be the underlying mechanism leading to genetic drift that might result in genetically different cell lines (Baker et al. 2016; Cai et al. 2014; Martins-Taylor and Xu 2012). When cell lines are used for protein production, stringent single cell cloning has been suggested as a favorable way to ensure cell identity and production quality. However, this was recently doubted as a study showed that also single-cell-clone-derived cell populations comprise variants of production efficiency and quality, up to the level of changes in copy number of transgene within the population (Ko et al. 2017).

Even though we confirmed some of the amino acid changing events (e.g. HSF1) that distinguished the LUHMES SP, none of those conclusively explained the observed differences in the toxicological response. The similar sensitivity to largely diverse toxicants suggests that fundamental apoptosis and stress pathways did not differ between the SP.

Bioinformatic data-mining revealed that genetic alterations are particularly frequent within pathways related to the dopaminergic phenotype. Intracellular dopamine has been clearly identified as factor promoting neuronal cell death (Mor et al. 2017; Mosharov et al. 2009). Therefore, the capacity of LUHMES to produce dopamine results in a selective pressure to accumulate mutations affecting their dopaminergic phenotype. Both SP seemed to have taken a different path to decrease potential dopamine toxicity. For “ATCC”, the down-regulation of tyrosine-hydroxylase (TH) is the most obviously reducing dopamine stress. Interestingly, this did not occur by inactivation of the TH gene, but rather by some upstream regulation. In the same vein, dopamine transporter (DAT) was affected indirectly, and in a way that was not obviously predicted by genome data.

Direct phenotypic characterization helped to identify differences in the dopaminergic phenotype of the SP. Especially, the lower expression and activity levels of the DAT provides a conclusive and sufficient explanation for the observed differences in the toxicological response to MPP^+^, as the toxicant requires this transporter (Schildknecht et al. 2015, 2017; Terron et al. 2018) to enter the cells. The toxicity we observed at higher concentrations of MPP^+^ (≥ 100 µM) in the “ATCC” SP might be mediated by low abundance of the DAT (down-regulated, but not totally deficient) or by other transporters, such as the organic cation transporter-3 (Cui et al. 2009; Schildknecht et al. 2015, 2017). The fact that both SP reacted similarly to rotenone, a toxicant with the same target as MPP^+^ (mitochondrial complex I), suggests that toxicokinetic (i.e. transport) factors were mainly responsible for the observed sensitivity difference with MPP^+^.

In summary, the data presented here provide an important example for gaps in the commonly used cell identification approaches, and for the need to reconsider good practices in the field (Leist et al. 2014; Pamies et al. 2017; Ramirez et al. 2013; Reid 2017; Stacey 2012, 2017). To ensure proper cell functionality, we suggest to include a “fit-for-purpose” phenotypic testing strategy in cell authentication procedures, and to define performance standards for desired cell functions. Examples for such a testing strategy could be (1) to include testing for dopamine transporter expression and functionality via measurement of MPP^+^ uptake when establishing the MPP^+^ model or (2) measurement of Aβ secretion when using the cells to model processing of the amyloid precursor protein (APP) (Scholz et al. 2018). Adoption of such standards would improve reproducibility of in vitro data and comparability across studies. Therefore, an important issue concerning a perceived reproducibility crisis of science (Freedman 2015; Freedman et al. 2015a, b) would be addressed. Future refinements can be envisaged already now, e.g. addressing heterogeneity and genetic mosaicism within given SP, for example by single cell sequencing (McConnell et al. 2013) or functional testing after subcloning.

## Electronic supplementary material

Below is the link to the electronic supplementary material.

Supplementary material 1 (PDF 1328 KB)
