# Supplementary material for: Major changes of cell function and toxicant sensitivity in cultured cells undergoing mild, quasi-natural genetic drift
Source: Arch Toxicol. 2018 Oct 8;92(12):3487–503. doi: 10.1007/s00204-018-2326-5 (PMC6290691; doi:10.1007/s00204-018-2326-5)
Supplement: Supplementary file 1 — Supplementary material 1 (PDF 1328 KB) [file 204_2018_2326_MOESM1_ESM.pdf]

Supplementary information:

Major changes of cell function and toxicant sensitivity in cultured cells undergoing mild, quasi-natural genetic drift

Simon Gutbier[1], Patrick May[2], Sylvie Berthelot[1], Abhimanyu Krishna[2], Timo Trefzer[1], Mehri Behbehani[1], Liudmila Efremova[1], Johannes Delp[1], Gerhard Gstraunthaler[3], Tanja Waldmann[1], and Marcel Leist[1].

[1]: Department for *in vitro* toxicology and biomedicine (Doerenkamp-Zbinden chair), University of Konstanz, Konstanz, Germany,
[2]:Luxembourg Centre for Systems Biomedicine, University of Luxembourg, Esch-sur-Alzette, Luxembourg,
[3]:Division of Physiology, Innsbruck Medical University, Schöpfstraße 41/1, A-6020 Innsbruck, Austria

Running title: Genetic drift of cultured cells

STR marker \ Source	This study		ATCC website
	UKN LUHMES	ATCC LUHMES	ATCC LUHMES
Amelogenin	X	X	X
CSF1PO	13,14	13,14	13,14
D5S818	11,13	11,13	11,13
D13S317	9,11	9,11	9,11
D7S820	11,13	11,13	11,13
D16S539	11,12	11,12	11,12
vWA	14,17	14,17	14,17
THO1	7,9.3	7,9.3	7,9.3
TPOX	8	8	8
Penta D	12,13	12,13	
D8S1179	12,13	12,13	
FGA	19,21	19,21	
D3S1358	17,18	17,18	
D21S11	30,31	30,31	
D18S51	12	12	
Penta E	11,13	11,13	

Fig. S1: Short tandem repeat (STR) profiles of LUHMES SP

Identity of both SP "UKN" and "ATCC" was confirmed by STR (Short Tandem Repeat) analysis. DNA samples from the SPs were prepared using a commercial kit (Puregene Cell Kit, Qiagen). The kit GlobalFiler® PCR Amplification Kit (Thermofisher) was then used to determine the cell-specific profile for 16 different genomic loci (left column). Results from SP used in this study are listed in the two centre columns. STR-profile provided by the ATCC website (https://www.lgcstandards-atcc.org/Products/All/CRL-2927.aspx#specifications) is listed in the left column.

A

Overview of structural variants (SV) detected			
	**Deletions**	**Duplications**	**Inversions**
**Total**	1726	273	435
Found in "DGV"	150	119	172
Overlap with "CG Baseline"	1706	252	128
**Post-filtering for known SVs**	**17**	**16**	**188**
Concordant	7	2	6
Only UKN	1	0	2
Only ATCC	9	14	179

B

Genes overlapping with SVs			
**Platform**	**Deletions**	**Duplications**	**Inversions**
Both platforms		RMDN1	
UKN			ADCY8, TMEM167A
ATCC	CSMD1, C8orf44-SGK3, , EFHD1, FAM209A, FAM209B, GCNT7, MIR512-1, MIR512-2, SGK3	AHRR, CHST9 , SLC12A7, SLC13A3, SMC1B, TCF25, VPS16, WDR27	ACTR3B, ADAMTS7, ANKRD36, ARHGEF7, ATP1B4, C14orf39, CCDC129, CD8B, CD97, CHRFAM7A, CHRNA7, CLYBL, CNTNAP5, CSF2RA, CYB5R4, DCK, DEFB107A, DEFB107B, DNM1P35, DNM1P46, DPP6, EMR2, ETFA, EVPL, EVPLL, EXOC6B, F11-AS1, F13A1, FAM184B, FAM65C, FAR2P1, FKBP14, GALNT13, GOLGA8A, GUSBP11, HERC2, HERC2P10, HERC2P9, KCNH5, KRTAP4-6, KRTAP4-7, LINC00607, LOC100288637, LOC101927437, LOC202181, LOC440910, LOC728554, MGLL, MIR4435-1HG, MOK, NOL10, NOMO1, NOMO2, NPSR1, NPSR1-AS1, NRXN1, OTUD7A, PDCD6IPP2, POTEF, RAD51B, RALYL, ROCK1, ROCK1P1, RPSAP58, SDHAP1, SDHAP2, SH3BP2, SIMC1, THOC3, TMEM207, TYRO3, TYW1, TYW1B, VCX, VCX2, ZGRF1, ZNF521, ZNF701, ZNF702P, ZNF91

Fig. S2: Filtering of SVs for location in protein-coding regions

A: Detected structural variants (SVs) were filtered using DGV and CG baseline databases and number of SP specific deletions, duplications and inversions was calculated. **B:** Detected SVs were tested for proximity to genes and possible affected genes were listed according to their occurrence (both SP or specific) and their property (deletion, duplication or inversion)

Source of identified CNV in protein coding regions		
**UKN**	**Both**	**ATCC**
ACVR1C, ADARB1, ARHGAP21, ATP10B, CAMK4, CMYA5, CPAMD8, EFCAB1, GSG1L, ITGA9, NWD1, PRKAR1B, RNF17, SH3GL3, SPOCK1, SUPT3H	C1R, DLGAP4, DOCK1, DPP6, GAGE12J, GAGE13, GAGE2A, GAGE2C, GAGE2E, GAGE8, GRK1, HMCN2, INPP5D, MID1, MLXIP, MUSK, NRG3, SH3RF3, TWIST2	ADAMTS20, APP, C12orf42, C12orf66, CCDC138, CDH8, CENPP, IGSF11, LINC00922, LOC100507065, LOC728554, MARK1, MGMT, MME, MMP16, PRKCA, SCRN3, TNKS, TSHR, TUBA1A, ZNF814 ANTXR2, CSAG3, CXorf51A, CXorf51B, FAM72C, FAM72D, LINC00457, MAGEA2, MAGEA2B, MAGEA6, XAGE2, NXF2, NXF2B, TCP11X2

Fig. S3: List of copy number variations
The whole genome sequencing data from LUHMES "ATCC" and "UKN" SP were mined for rare LUHMES specific copy number variations (CNV). They were filtered for those overlapping protein-coding genes. Those that were found to overlap with the same gene in both SP were identified (middle column). Gains in copy number are marked in blue and losses in copy number are marked in red. Notably, the latter group comprises partial and heterozygous (single-allelic) losses (i.e. not necessarily 'knock-outs')

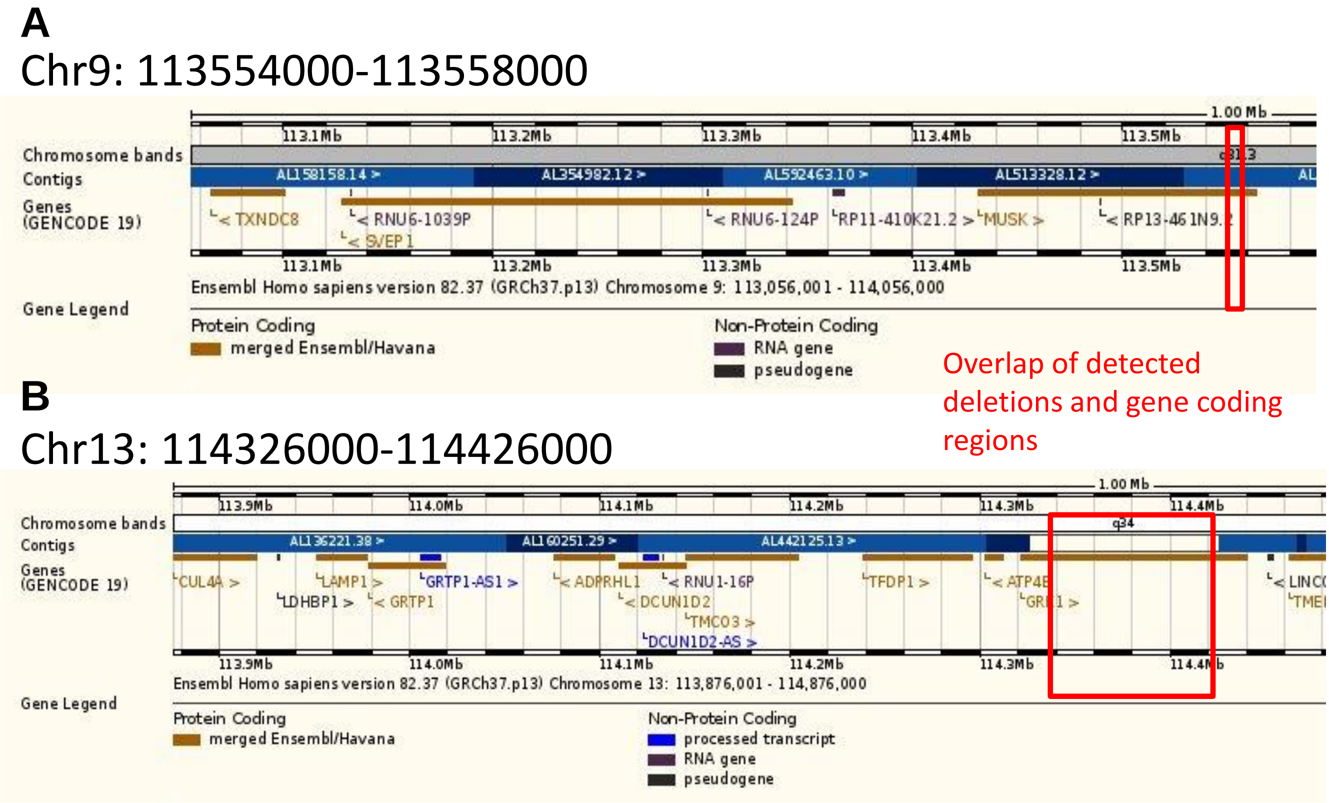

Fig. S4: Examples of CNVs

Examples for filtered copy number variations (CNVs) marked in Fig. 2 and their location in the genome. Pictures were generated using the Ensembl genome browser 82.37 (https://www.ensembl.org/) with the human genome assembly GRCh37. Coordinates of detected CNVs were entered into the genome browser and graphical depiction of the region of the corresponding chromosome was used to show where in the gene the CNV is located (red boxes). **A:** CNV of a size of 4 kbp in the Muscle Associated Receptor Tyrosine Kinase (MUSK) gene located on chromosome 9. **B:** CNV of a size of 100 kbp in the G Protein-Coupled Receptor Kinase 1 (GRK1) gene located on chromosome 13.

A:

Single nucleotide variants (SNVs)	
**Database overlap**	**Detected in one or both platforms**
**Total variants**	4 509 102
Exome Sequencing Project	43 160
1000 Genome Project	3 936 535
Complete Genomics 69 Genomes Baseline	3 749 949
dbSNP 138	4 182 029
avSNP	4 235 576
Found in one of the above databases	4 346 399
**Somatic variants**	**162 703**
**Somatic variants after platform bias correction**	**46800**

B

Somatic filtered variants			
**Variant type**	**Concordant**	**discordant**	**partial**
**deletion**	1105	2161	4
**insertion**	747	3828	7
**SNV**	12131	1916	10
**substitution**	421	1067	3
**sum**	14404	8972	24

C

Somatic filtered amino acid changing variants			
**Variant type**	**concordant**	**discordant**	**partial**
**deletion**	1	1	0
**insertion**	0	21	0
**SNV**	29	6	0
**substitution**	3	1	0
**sum**	33	29	0

Fig. S5: Genomic differences- prediction of affected genes

A: Detected SNV found in both or only one of the SP were filtered against SNV found in the indicated databases in order to reduce the number and identify SP specific SNVs (somatic variants) **B:** List of filtered and platform bias corrected somatic variants. After filtering detected SNV against known databases (Fig. S6A), platform corrected SNVs were grouped into concordant (occurring in both SP), discordant (occurring only in one SP) and partial overlapping SNVs. **C:** Filtered SNVs obtained in B were tested for their potential to change the amino acid of a protein. The identified proteins are listed in Fig. 3A.

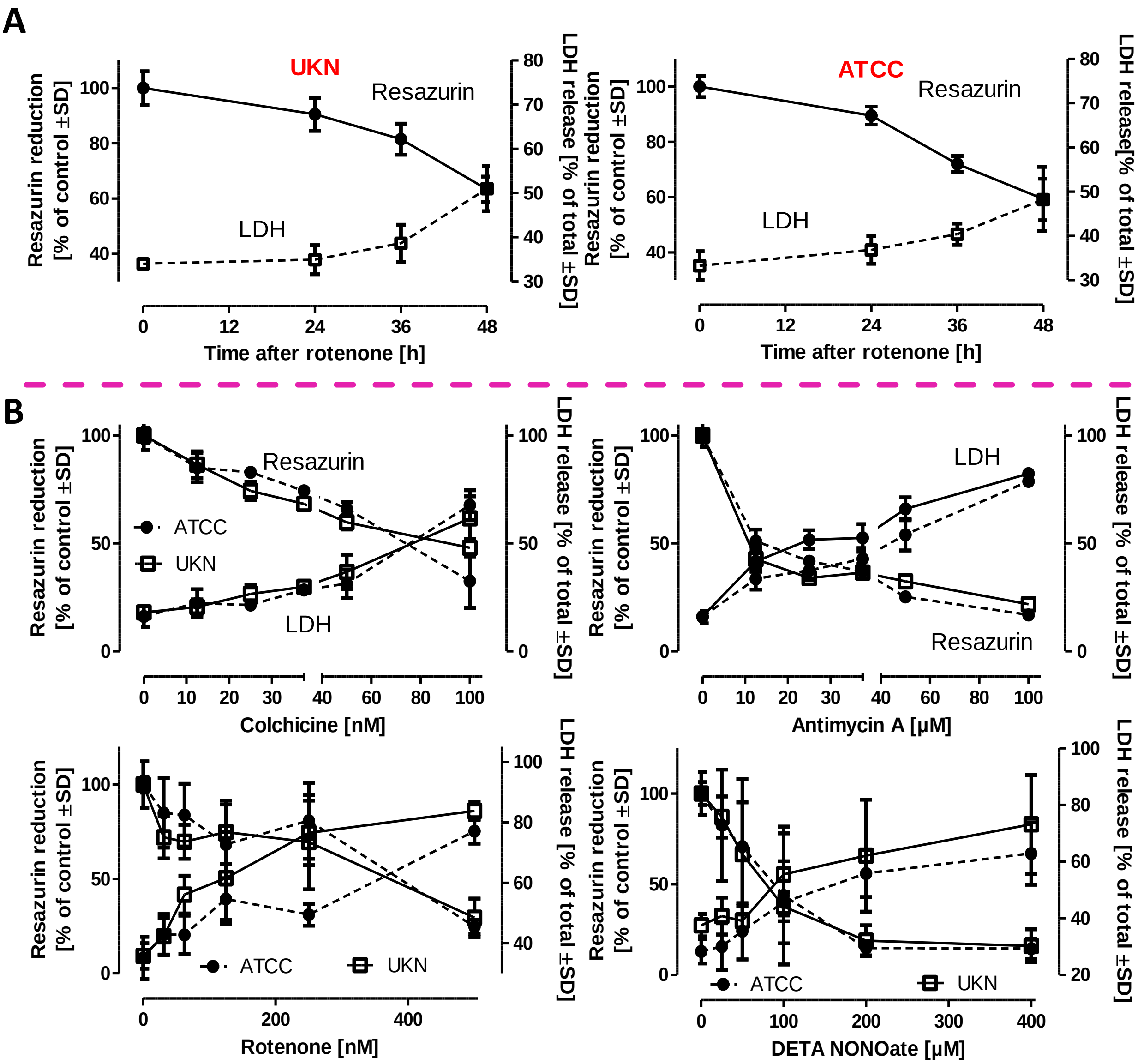

Fig. S6: Similar and different concentration response examples in UKN and ATCC LUHMES

LUHMES cell subpopulations (SP) from two different sources (University of Konstanz (UKN); American Type Culture Collection (ATCC)) were cultured according to a standard protocol. Cells were seeded at a density of $1.5x10^5$ cells/cm^2 at day (d2). Medium was exchanged at d4. Toxicant exposure started at d6. **A: "**UKN" and "ATCC" LUHMES were incubated with 750 nM rotenone for the indicated time periods. After incubation, viability was assessed measuring resazurin reduction and LDH release **B:** "UKN" and "ATCC" SP were incubated with different concentrations of colchicine, antimycin A, rotenone and DETA-NONOate for 48 h. After incubation, viability was assessed by measuring resazurin reduction and LDH release. Data are means ± SD of three independent experiments.

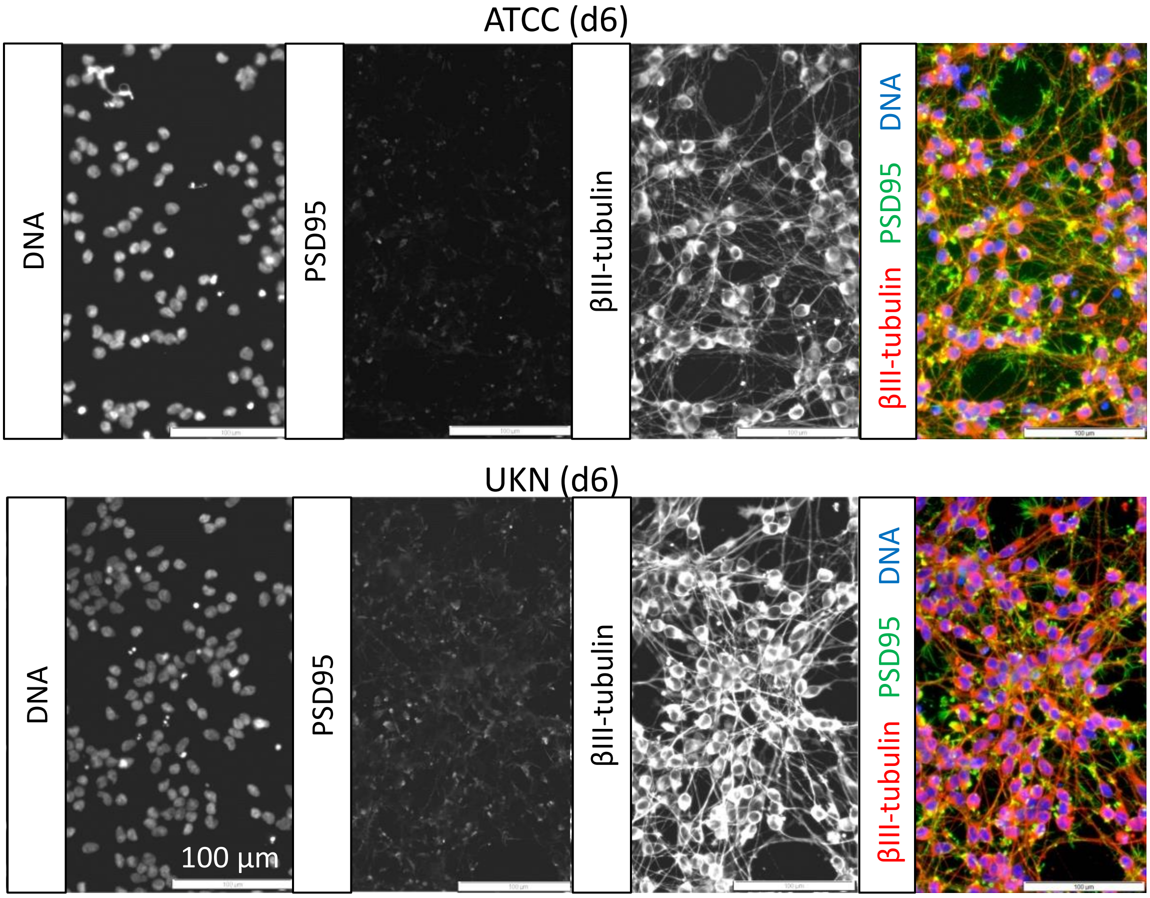

Fig. S7: Phenotypic similarities of LUHMES SP

LUHMES cells from two different sources (UKN and ATCC) were cultured and differentiated according to a published standard protocol. Differentiated (d6) LUHMES cells at a density of 1.5×10^5 cells/cm^2 were stained for post synaptic density protein 95 (PSD95) and β-III-tubulin and morphological properties were examined by fluorescence microscopy using a Olympus IX81 inverted epifluorescence microscope with a 20x objective.

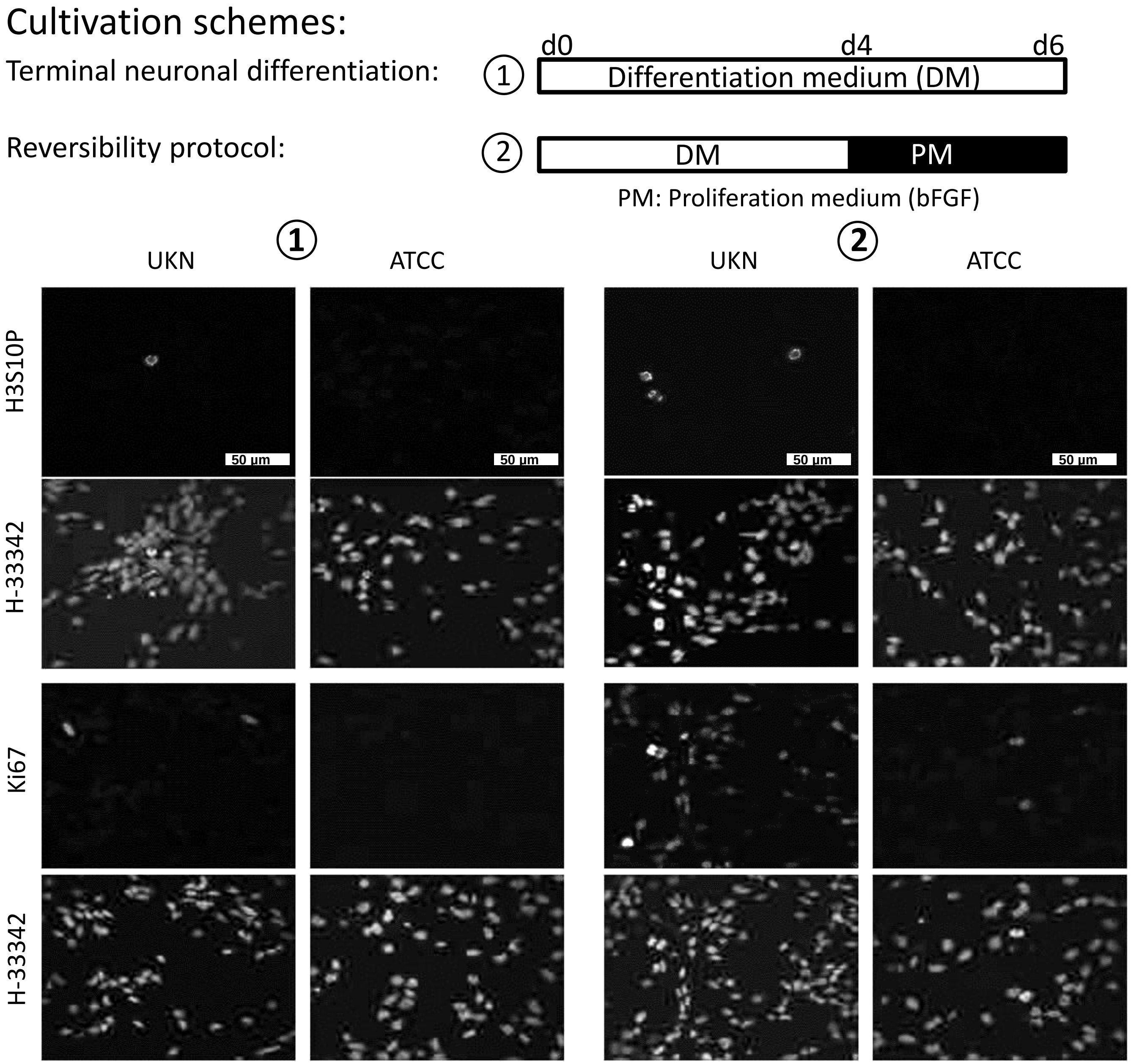

Fig. S8: Cell cycle exit

Two different SP of LUHMES cells “UKN” and “ATCC” were cultured according to a standard protocol until d2 and then replated at a density of $1.5x10^5$ cells/cm^2. d2 LUHMES cells were cultured in to different cultivation procedures. Therefore medium was exchanged at d4 to either 1) differentiation medium (DM) or 2) proliferation medium (PM). Differentiated (d6) LUHMES cells were stained for histone 3 serine 10 phosphorylation (H3S10P) or Proliferation-related Ki67 Antigen (Ki67) and positive nuclei were examined in comparison to total nuclei count by fluorescence microscopy

Ingenuity Analysis of differential mutations (CNV/SV/SNV)

A **UKN**

Top Canonical Pathways		
Name	p-value	Overlap
Serotonin Receptor Signaling	1.22E-06	34.1 % 15/44
cAMP-mediated signaling	5.46E-04	15.1 % 33/219
Dopamine Receptor Signaling	5.47E-04	20.5 % 16/78
Tetrahydrobiopterin Biosynthesis I	5.62E-04	100.0 % 3/3
Tetrahydrobiopterin Biosynthesis II	5.62E-04	100.0 % 3/3

B **ATCC**

Top Canonical Pathways		
Name	p-value	Overlap
Breast Cancer Regulation by Stathmin1	6.31E-07	14.7 % 28/191
Role of NFAT in Cardiac Hypertrophy	6.00E-06	14.0 % 25/179
Protein Kinase A Signaling	5.44E-05	10.1 % 39/386
Cholecystokinin/Gastrin-mediated Signaling	2.12E-04	14.9 % 15/101
Calcium-induced T Lymphocyte Apoptosis	4.05E-04	17.2 % 11/64

Fig. S9: Top canonical pathways from ingenuity pathway analysis
For identification of SP specific pathways, possibly affected by genetic alterations, all genes affected by any SP-specific CNV, SV or SNV were used to test for pathway overrepresentation. Therefore, identified and filtered variants were analyzed by Ingenuity Pathway Analysis software (IPA®, *https://analysis.ingenuity.com/*). **A:** Top 5 canonical pathways for "UKN" LUHMES, pathways related to dopaminergic phenotype marked with red box. **B:** Top 5 canonical pathways for "ATCC" LUHMES, pathway related to dopaminergic phenotype marked with red box.

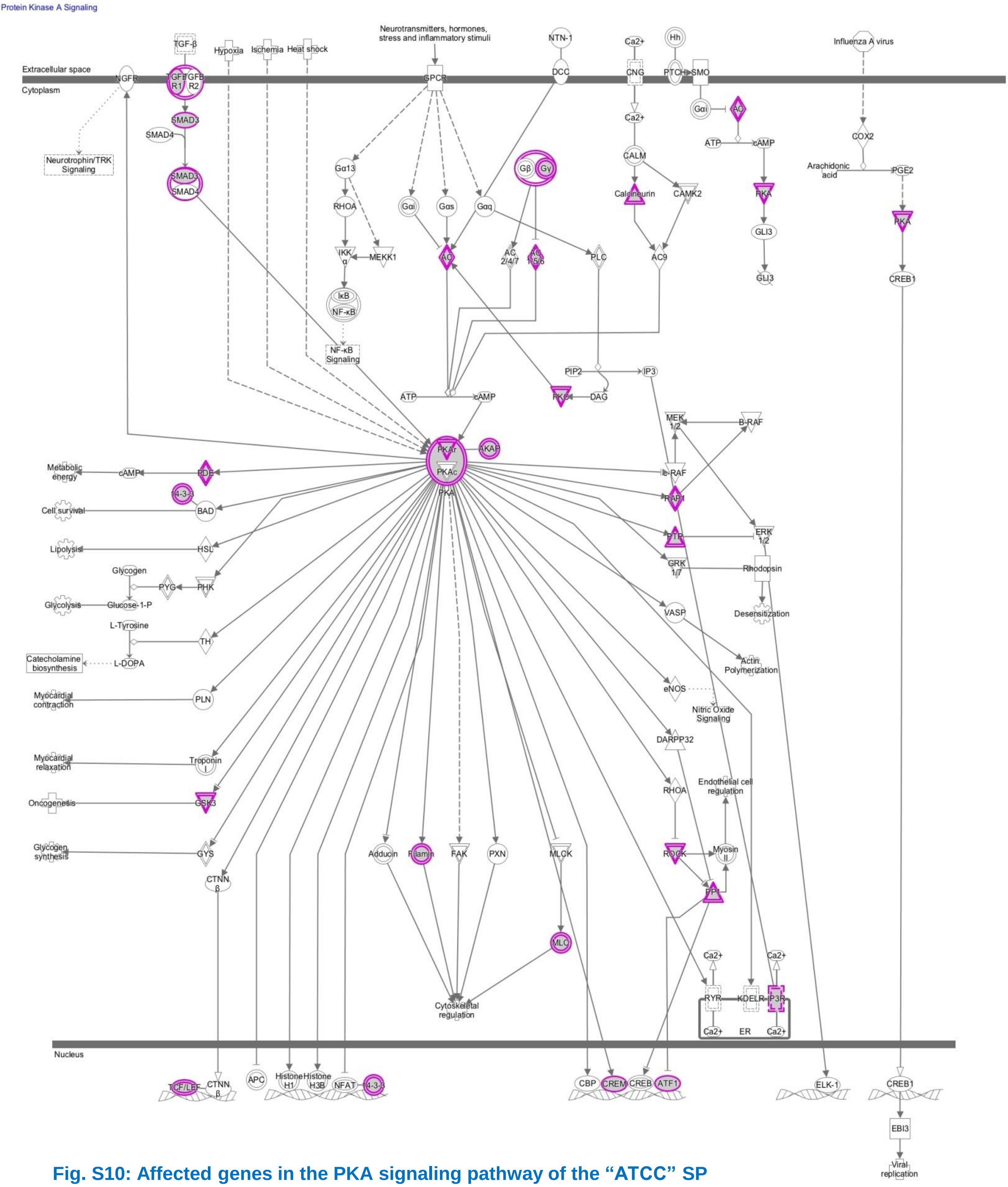

Fig. S10: Affected genes in the PKA signaling pathway of the "ATCC" SP

Pathway map of the PKA signaling retrieved from the Ingenuity platform. Proteins within this pathway possibly affected by a variant (CNV, SV and SNV) in the "ATCC" SP are circled in purple.

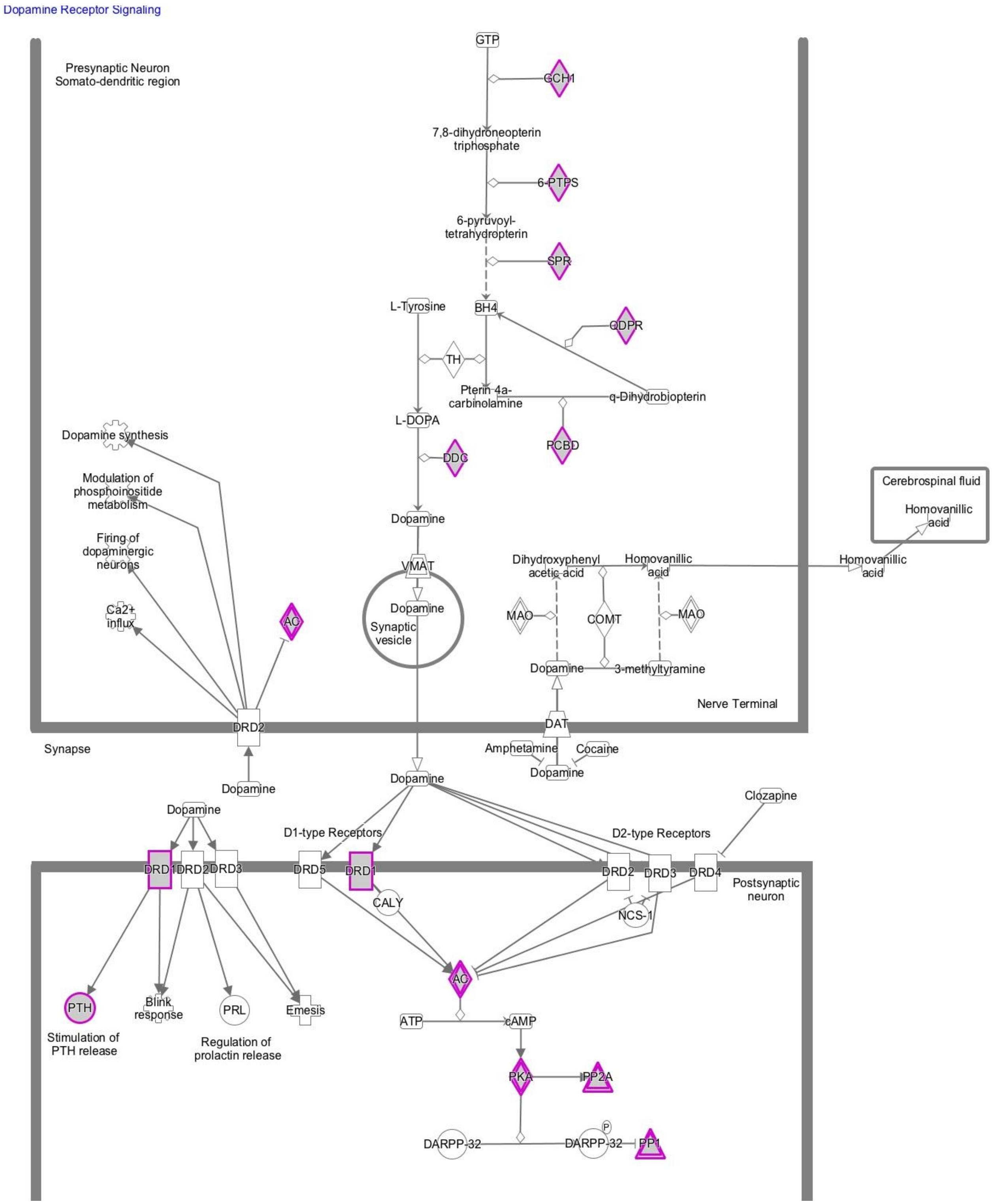

Fig. S11: Affected genes in the dopamine receptor signaling pathway of the "UKN" SP

Pathway map of the dopamine receptor signaling pathway retrieved from the Ingenuity platform. Proteins within this pathway possibly affected by a variant (CNV, SV and SNV) in the "UKN" SP are circled in purple.

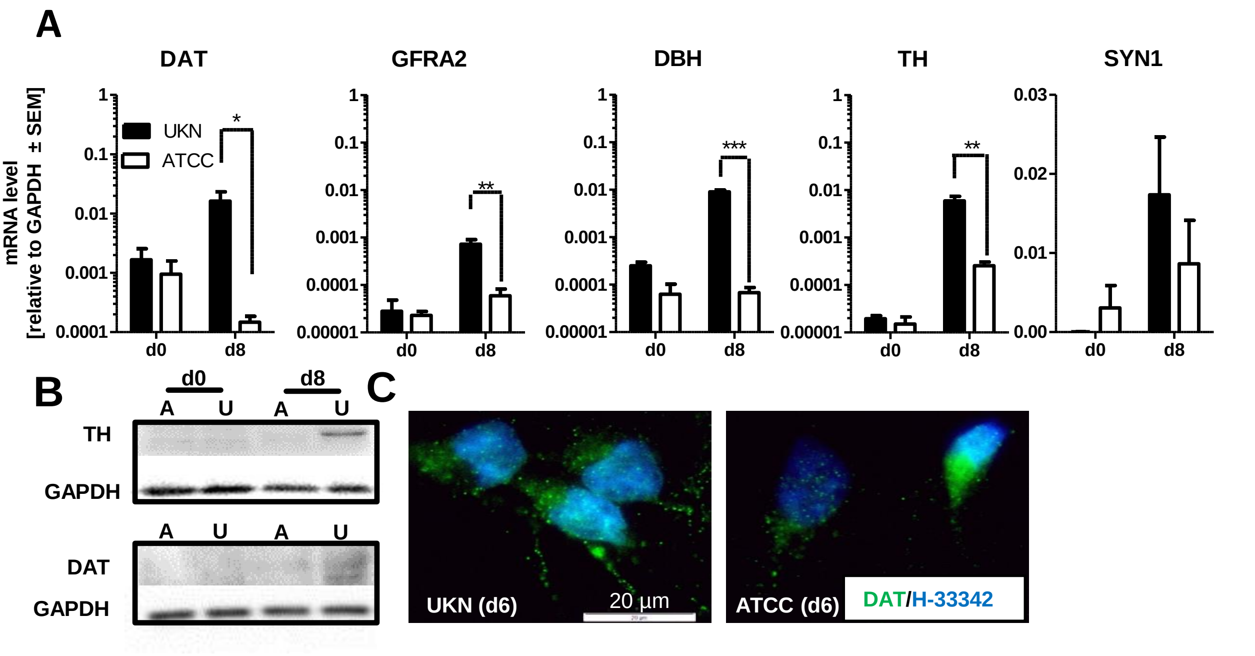

Fig. S12: Differentially expressed functional markers in the two LUHMES SP

Two different SP of LUHMES cells "UKN" and "ATCC" were cultured according to a standard protocol until d2 and than replated at a density of $1.5x10^5$ cells/cm^2. **A:** Changes in gene expression of the neurodevelopmental marker genes DAT, GFRA2, DBH, TH and SYN1 were compared at d0 and d8 of differentiation of LUHMES cells using qPCR. Values are expressed relative to expression levels in undifferentiated LUHMES cells and represent means ± SEM from three independent experiments. ***: p<0.001, **: p<0.01, *: p<0.5. **B:** Undifferentiated (d0) and mature (d8) LUHMES cells were lysed and analysed by Western blot using anti-TH (tyrosine hydroxylase), anti-DAT (dopamine transporter) and anti-GAPDH antibodies. **C:** Distribution and abundance of dopamine transporter in both SP was assessed by immunocytochemistry staining for dopamine transporter (DAT) and H-33342.

Antibodies used in this study				
**product**	**host**	**dilution**	**supplier**	**Cat. No.**
Dopamine transporter antibody	rat	1:1000	Millipore	MAB369
GAPDH antibody	mouse	1:10000	Invitrogen	39-8600
Monoclonal MAP2 antibody, IgG1	mouse	1:500	Sigma	M9942
Monoclonal Nestin antibbody, IgG1	mouse	1:500	R & D systems	MAB1259
Monoclonal Synapsin 1	mouse	1:500	Synaptic Systems	106 011
Monoclonal Tyrosine hydroxylase antibody, IgG1	mouse	1:500	Sigma	T2928
Monoclonal β-Tubulin (TUJ1) antibody	mouse	1:500	Covance	MMS-435P-250
Polyclonal GAD1 antibody	rabbit	1:500	Synaptic Systems	198003
Polyclonal PSD-95 antibody	rabbit	1:500	Invitrogen	51-6900
HSP70 (6B3)	rat	1:1000	Cell Signaling	4873
HSF1	rabbit	1:1000	Cell Signaling	4356
SirT6 (D8D12)	rabbit	1:1000	Cell Signaling	12486
NRXN3	rabbit	1:1000	Sigma	SAB1305223
Ki67	mouse	1:200	BD Bioscience	5136525X
CCNA1	mouse	1:200	Santa Cruz	Sc-271682
H3S10P	rabbit	1:200	Cell Signaling	9701
Alexa Fluor 488, IgG1 (γ1), anti mouse	goat	1:1000	Invitrogen	A21121
Alexa Fluor 555, IgG2a (γ2a), anti mouse	goat	1:1000	Invitrogen	A21137
Alexa Fluor 488, IgG anti-rabbit	chicken	1:1000	Invitrogen	A21441
Peroxidase conjugated, anti Mouse, IgG	goat	1:5000	Jackson Immuno Research	115-035-174
ECL Anti Rabbit, IgG	donkey	1:5000	GE Healthcare	NA934V
ECL Anti Rat, IgG	goat	1:5000	GE Healthcare	NA935

Supplementary table 1: Antibodies

Primers used in this study		
**qPCR (mRNA)**	**Forward**	**Reverse**
ACHE	5'-CTT CCT CCC CAA ATT GCT C-3'	5'-TCC AGT GCA CCA TGT AGG AG-3
DBH	5'-GCC TTC ATC CTC ACT GGC TA-3'	5'-GAC CAC CTT TCT CCC AGT CA-3'
DAT	5'-TGA CTT CTA CCG GCT CTG CG-3'	5'-AGA AGA CGA CGA AGC CGG AG-3'
DRD2	5'-GCC GGG TTG GCA ATG ATG CA-3'	5'-ACG GCG AGC ATC CTG AAC TT-3'
GAPDH	5'-CAC CAT CTT CCA GGA GCG AGA TC-3'	5'-GCA GGA GGC ATT GCT GAT GAT C-3'
GFRA1	5'-TGG AGG ATT CCC CAT ATG AA-3'	5'-TTG TTC CCT TTG GGA ATG TG-3'
GFRA2	5'-GAG TCA CTG GTG CGC CAG GA-3'	5'-GAG TCA CTG GTG CGC CAG GA-3'
GRIN1	5'-GAG AAG GTG CTG CAG TTT GA-3'	5'-GGT ATA CAG TGG CAG CAT CG-3'
HES5	5'-TTG GAG TTG GGC TGG TG-3'	5'-CCC AAA GAG AAA AAC CGA-3'
SNAP25	5'-CTG TCT TTC CTT CCC TCC CT-3'	5'-GGG TCA GTG ACG GGT TTG-3'
SYN1	5'-TCA GAC CTT CTA CCC CAA TCA-3'	5'-GTC CTG GAA GTC ATG CTG GT-3'
SYP	5'-CGA GGT CGA GTT CGA GTA CC-3'	5'-AAT TCG GCT GAC GAG GAG TA-3'
TH	5'-GCG CAG GAA GCT GAT TGC TG-3'	5'-TGT CTT CCC GGT AGC CGC TG-3'
TUBB3	5'-CGC CCA GTA TGA GGG AGA T-3'	5'-AGT CGC CCA CGT AGT TGC-3'
v-myc	5'-TTT TGG TCT CCA GGC TCT TC-3'	5'-GGAGTCCTTTGTGGGTTCAG-3'
VMAT2	5'-TGG GGA GGT GGC TTT GTG CT-3'	5'-CCC ATA GAC GGA CAC GTG CC-3'
BMP7	5'-GCC CCT CAG TCC CTG TAT C-3'	5'-GTG GTG AGT GGG GAG AGG T-3'
CCNA2	5'-GTT GCC CAG CCT TTA GCT C-3'	5'-GTT GCC CAG CCT TTA GCT C-3'
CCNB1	5'-GTC GCT GAG CTT CAG TTC CT-3'	5'-CTA CGG TAG CAG CAA TAA TAT AGT TCA-3'
CCND1	5'-TCC CTC CTA GCT GTC CTC CT-3'	5'-CGG ACT GCT TCT CTC CAA AC-3'
KIF14	5'-TTT GCC CCC AAG TTA ATC AT-3	5'-TCT CAC AAG ATT TTC AGG ACT GTT-3'
SHH	5'-ATG CTG CTG CTG GCG AGA T-3'	5'-CTG CTT GTA GGC TAA AGG GGT CA-3'

Supplementary table 2: Primers

Overview of sequencing data and genome status		
**Metric**	**UKN**	**ATCC**
Genome reference	37	
Gene annotations	37.2	
Fully called genome fraction	0,966	0,9643
Average coverage	33.25X	38.85X
Genome fraction where weightSumSequenceCoverage >= 5x	0,993	0,9966
Genome fraction where weightSumSequenceCoverage >= 10x	0,983	0,9947
Genome fraction where weightSumSequenceCoverage >= 20x	0,931	0,9871
Gender	female	female
SNP total count	3343761	3580196
SNP heterozygous/homozygous ratio	1,609	1,56
SNP transitions/transversions ratio	2,141	2,08
INS total count	217875	331630
DEL total count	222490	337441
SNP total count	21118	22085
INS total count	234	325
DEL total count	242	364
Synonymous SNP loci	10311	10641
Non-synonymous SNP loci	9487	11433

Supplementary table 3: Overview of sequencing data and genome status
